# Knitted Pneumatic Fabrics for Dynamic Pressure Modulation in Personalized Healthcare Wearables

**DOI:** 10.1002/advs.202518305

**Published:** 2026-03-27

**Authors:** Xiaoyu Chen, Jintu Fan, Rong Zheng, Qing Chen, Fengxin Sun, Huanhuan Liu

**Affiliations:** ^1^ College of Fashion and Design Donghua University Shanghai China; ^2^ School of Fashion and Textiles The Hong Kong Polytechnic University Hong Kong China; ^3^ Research Centre of Textiles for Future Fashion and School of Fashion and Textiles PolyU Xingguo Technology and Innovation Research Institute Hong Kong China; ^4^ Shanghai International Fashion Innovation Center Donghua University Shanghai China; ^5^ Laboratory of Soft Fibrous Materials & Physics Jiangnan University Wuxi China

**Keywords:** dynamic pressure modulation, knitted pneumatic fabrics, medical gradient compression, personalized healthcare wearables, pneumatic fibers

## Abstract

Wearable pneumatic textiles hold significant potential for healthcare applications, particularly in preventing and treating stress‐related injuries and diseases. Current pneumatic healthcare wearables often suffer from undesired multidirectional expansion of air chambers, poor conformability to complex body contours, and difficulty in achieving controllable pressure gradients required for medical applications. To overcome these issues, herein, a Pneumatic Pressure‐Regulating Knitted Fabric (PPKF) was developed. Its design utilizes a knitted pneumatic circuit to seamlessly integrate an ultralow‐expansion pneumatic fiber within a dual‐channel structure. Ultralow radial expansion (≤ 1.41%) of pneumatic fiber enables controlled contractile deformation when embedded within knit structures. By strategically modulating channel spacing, PPKF achieves dynamic pressure modulation, delivering adjustable pressures (3–32 kPa) and clinically relevant gradient compression ratios (100.0%, 77.6%, 51.8%, 31.0%, 14.6%). PPKF‐based garments exhibit superior wearability, including breathability (949.56 mm/s), excellent fit, and minimal deformation (≤  5.2%), outperforming commercially available alternatives. The integrated PPKF manufacturing strategy establishes a structure‐driven paradigm, providing new design principles for pressure‐regulating knitted textiles with enhanced wearability and multifunctionality. Substantial implementation potential is demonstrated across medical rehabilitation, aerospace systems, and athletic protection.

## Graphical abstract

A pneumatic pressure‐regulating knitted fabric (PPKF) is developed, in which knitted pneumatic circuit enables the seamless integration of ultralow radial expansion pneumatic fiber into dual‐channel textile structure. By strategically modulating channel spacing, delivering adjustable dynamic pressures and clinically relevant gradient compression ratios. The PPKF demonstrates scalable design principles for healthcare wearable requiring wearability and multifunctional.

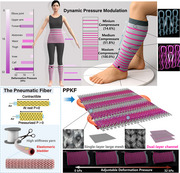

## 1 Introduction

Venous blood flow progresses from higher‐pressure distal extremities toward lower‐pressure proximal regions via pressure gradients. In varicose veins, valvular incompetence obstructs venous return, elevating distal venous pressure [1]. Compression garments mimic the muscle pump mechanism by applying external gradient pressure, achieving both preventive and therapeutic effects [2, 3]. As a critical parameter in compression therapy [4, 5], gradient pressure typically decreases [6] or increases [7] along anatomical axis (distal to proximal). Current pneumatic medical garments predominantly employ sealed inflatable bag, yet their tendency to delaminate from the skin surface compromises pressure transfer efficiency, resulting in edge force dissipation and non‐uniform compression [8] (Figure S1). Furthermore, most designs require multiple pneumatic supply lines connecting discrete pressure zones, increasing manufacturing complexity and cost.

Textiles are increasingly used as substrates for programmable active systems, particularly in actuated textiles [9, 10, 11]. Fluid‐driven technologies, especially gases, are prominent in active pressurized garments due to their compliance, accessibility, and rapid response [12, 13]. Fluid‐induced stress transmission integrates easily into soft media, generating quasi‐static displacement [14], shear [15], compression [16], and relatively high forces [17]. This facilitates applications in adaptive assistive dressing [18], movement assistance [19], haptic interaction [20, 21], and compression therapy [22, 23, 24]. However, traditional pneumatic compression garments rely on sealed air chambers [11, 24], which cause excessive garment expansion (increased bulk, restricted movement [25]) and poor fit due to mismatched body contours [26]. Therefore, combining soft textile materials with air‐driven technology is crucial for developing wearable active compression garments.

To overcome the above‐mentioned drawbacks, knitted fabrics offer potential for controllable compression garments [27]. Their inherent structure of multiple unit loops provides excellent breathability [28], elasticity [29], and tensile strength [30], making them ideal textile materials. Knitted fabrics present two key advantages for active compression applications: rapid machine production compared to cut‐and‐sewn garments, and the ability to incorporate active materials for programmable functions [31]. Local mechanical properties can be precisely controlled by altering yarn [32], pattern [33], and stitching [34, 35] in specific areas. Furthermore, knitted fabrics can form 3D shapes [36, 37] to conform to body contours, enhancing fit of garments [27]. Therefore, utilizing knit structures holds significant practical promise for developing active pressure‐regulating clothing.

Seamless integration of fiber‐level actuators within textiles enables programmable deformation control (e.g., bending [38], coiling [39], contracting [40], extending [41]), establishing this approach as an emerging paradigm for advanced wearable design. However, pneumatic artificial muscles (PAM) [42, 43, 44, 45, 46], as common actuators, often face challenges when integrated with textiles. Their large diameter (10–30 mm when unpressurized) causes excessive deformation and expansion [47, 48], leading to poor fit of garments. Currently, minimal research has explored knitted structures for controllable dynamic gradient pressurization, particularly by integrating functional pneumatic fibers to regulate pressure.

**FIGURE 1:**
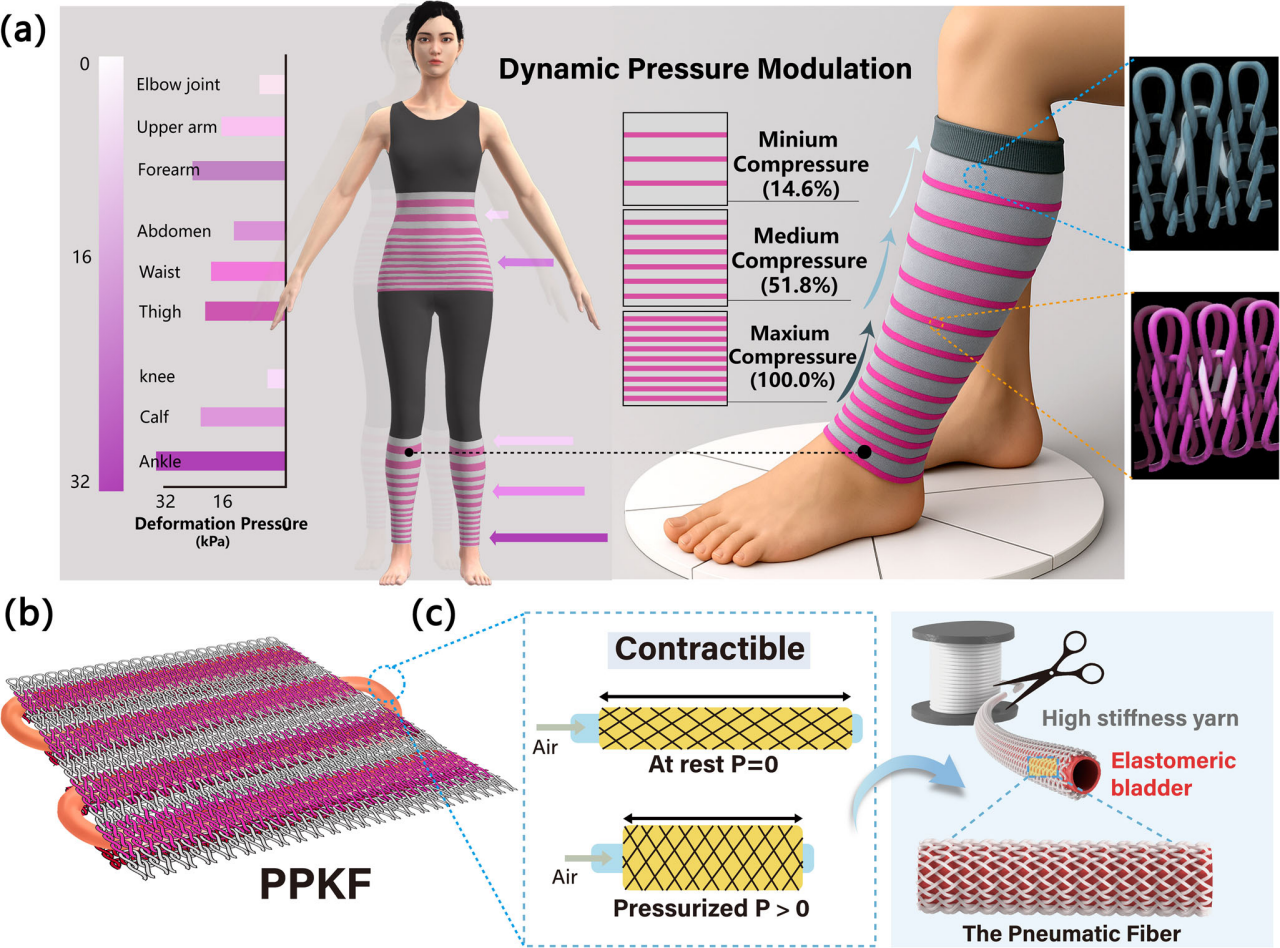
The PPKF with dynamic pressure modulation. (a) Dynamic pressure modulation for personalized healthcare wearables, which enables spatially gradient and adjustable pressure distribution. (b) The knitted pneumatic circuit in PPKF, seamless integration of an ultralow‐expansion pneumatic fiber through dual‐channel knitting structure. (c) Pneumatic fiber design inspired by PAM that enables controllable axial contraction.

Here, for the first time, we develop a pneumatic pressure‐regulating knitted fabric (PPKF) using a pneumatic fiber and dual‐channel knitting structure that shows closed‐loop strain control, dynamic pressure modulation, and miniaturization for seamless textile integration in a single package (Figure 1a,b). These are popular in research of soft actuators and McKibben [42], but are novel for knitted pneumatic compression healthcare wearables. Driven by external air pressure, the fiber produces controllable axial contraction, mimicking human muscle movement (Figure 1c; Movie S1). Low radial expansion and high contraction are achieved by adjusting the braiding angle and yarn material. Knitted structure design forms multiple hollow channels on the fabric surface, seamlessly integrating the pneumatic fiber. Combined with yarn characteristics, this maximizes conversion of fiber axial contraction into effective clothing pressure. Dynamic pressure modulation is achieved by modifying channel spacing via knitting pattern adjustments. The PPKF excels at high power levels, exhibiting low radial expansion to minimize volume changes during deformation, superior breathability, and an adjustable pressure range (3–32 kPa) with a gradient ratio suitable for medical compression garment. This research contributes to the practical application of soft‐driven textiles and advances clinically personalized healthcare wearables.

## 2 Results and Discussion

### 2.1 Structure Design and Manufacturing

**FIGURE 2:**
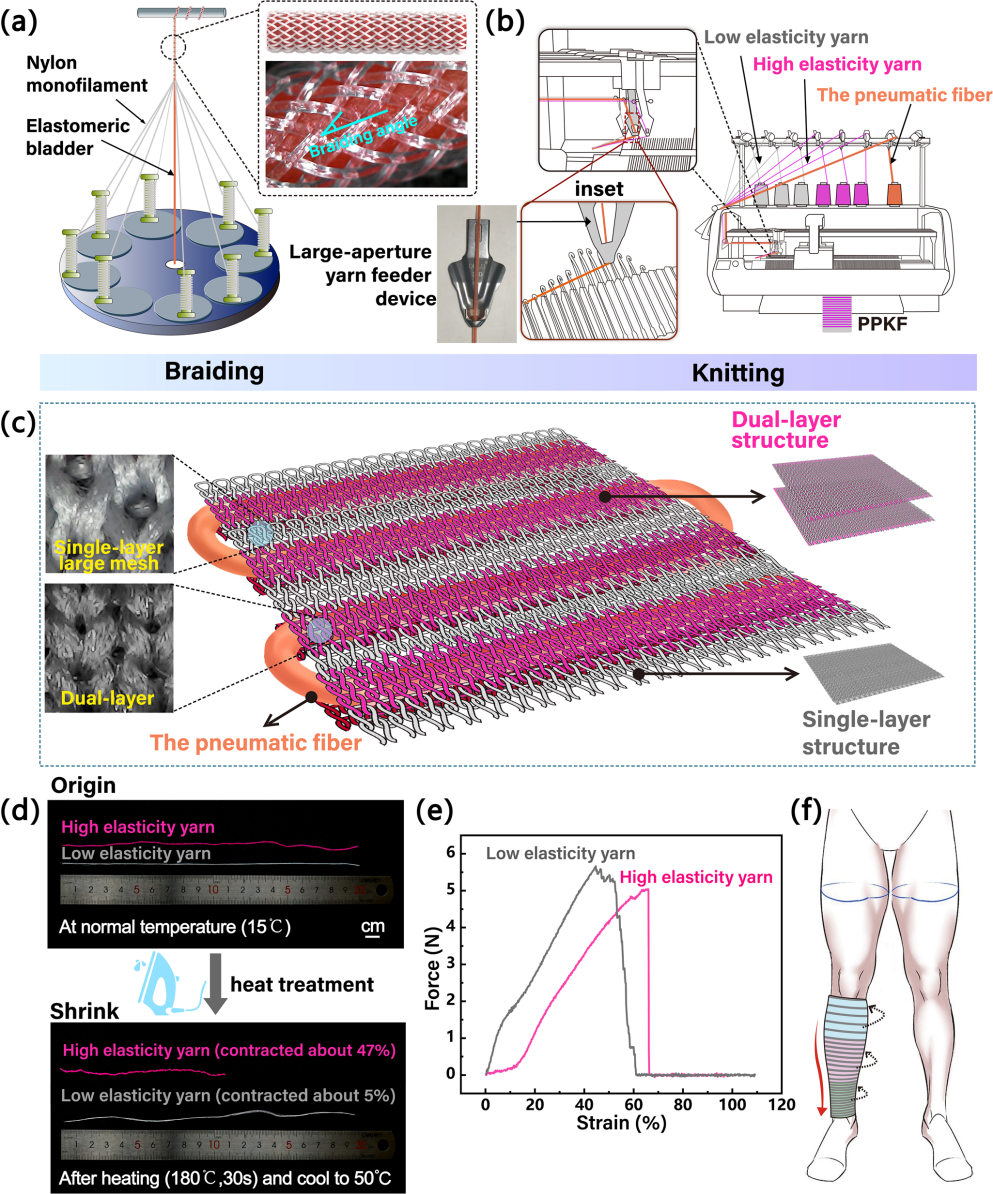
Fabrication and design of pneumatic fibers and PPKF. (a) The pneumatic fiber manufacturing process schematic. (b) Fabrication of the PPKF. (c) Structural schematic of the PPKF. (d) The high elasticity fiber curls after heating and contracts to about 47% of its original length. (e) Mechanical comparison of high elasticity yarn and low elasticity yarn. (f) The fit of PPKF and customizable gradient compression.

To address limitations and balance radial expansion, wearability, and adjustable gradient pressurization in pneumatic knitted fabric, the PPKF consists of pneumatic fibers and a knitted structure layer. Inspired by PAM, each pneumatic fiber has an outer braid mesh and an inner elastomeric bladder. When under pressure, the inner bladder expands, causing the outer mesh to expand radially. This braid structure then converts the radial expansion into axial contraction [43]. Modern braiding technology enables radially uniform expansion of the pneumatic fiber during integrated manufacturing (Figure 2a; Figure S2). To maximize cost‐effectiveness, we used the thinnest commercially available elastomeric bladder and selected smooth nylon monofilament for the outer mesh to minimize deformation friction. Furthermore, the difference in stiffness between the elastomeric bladder and the nylon monofilament (Figure S3) means that the stiff nylon monofilament resists deformation under pressure, maximizing the conversion of fiber axial contraction into contractile force.

The yarn feeder aperture was enlarged to accommodate the pneumatic fiber diameters, enabling their integration as active yarns in knitted textiles via digital knitting machine while maintaining structural integrity (Figure 2b; Figure S4). The design incorporates channel zones with hollow‐tube structures and non channel zones. The channel area employs a dual‐layer structure to embed the pneumatic fiber, while the non‐channel area uses a single‐layer structure with larger mesh for breathability (Figure 2c).

Elastic polyester, known for its extensibility, comfort, and common use in compression garments [14]. Further elucidate the deformation mechanism of knitted fabrics. During the knitting process, fabrics are frequently subjected to various uneven external forces (including twisting, stretching, and pulling), leading to alterations in both the internal yarn structure and external morphology. This results in dimensional instability (size and shape) and inconsistent mechanical properties, thereby compromising the fabric's wearability. Therefore, the PPKF must undergo heat setting treatment [49]. During the heat setting process of elastic polyester fabrics, molecular chains in the amorphous regions undergo orientation relaxation, leading to crimp formation [50]. The crimp shrinkage rate of elastic fibers is influenced by the elasticity of the filament bundle, a higher shrinkage rate indicates greater elasticity of yarn [51]. The heat setting temperature for polyester knits typically ranges from 180 to 210°C, with a setting time of 20 to 90 s [52].

Following this standard, we subjected two polyester yarns with differing elasticities to thermal treatment. Interestingly, we observed that high elasticity yarns exhibited a greater shrinkage rate than low elasticity yarns during heat setting (Figure 2d). This finding aligns with the conclusion that low elasticity yarns demonstrate lower shrinkage after heat treatment due to their high elastic modulus (Figure 2e) [53]. Therefore, this characteristic makes high elasticity yarn suitable for the PPKF channel area. During fabric heat treatment, yarn shrinkage causes the channels to wrap more tightly around the embedded pneumatic fibers, thereby increasing friction between the pneumatic fibers and surrounding yarns during deformation. This reduces deformation slippage and effectively drives the deformation of the knitted fabric. Simultaneously, low elasticity yarns maintain excellent strength after heat‐ treatment due to their higher elastic modulus [53]. These yarns are suitable for non‐channel areas, preventing excessive deformation during fabric heating while stabilizing PPKF dimensions.

The modulating properties of knitted fabrics enable versatile structural design. The PPKF can dynamically adjust stitch counts during knitting, allowing garment patterns to adapt to human body contours (Figure 2f). By strategically varying single‐layer row counts, the density of dual‐layer channels is precisely modulated to achieve dynamic pressure modulation. This spatial control establishes distinct pressurization zones at predetermined distances. Through seamless integration of the pneumatic fiber as functional yarns, the PPKF demonstrates significant potential for garment miniaturization, customizable gradient compression, and enhanced wearability. Further details can be found in the next section.

### 2.2 Wearable Performance of the PPKF

**FIGURE 3:**
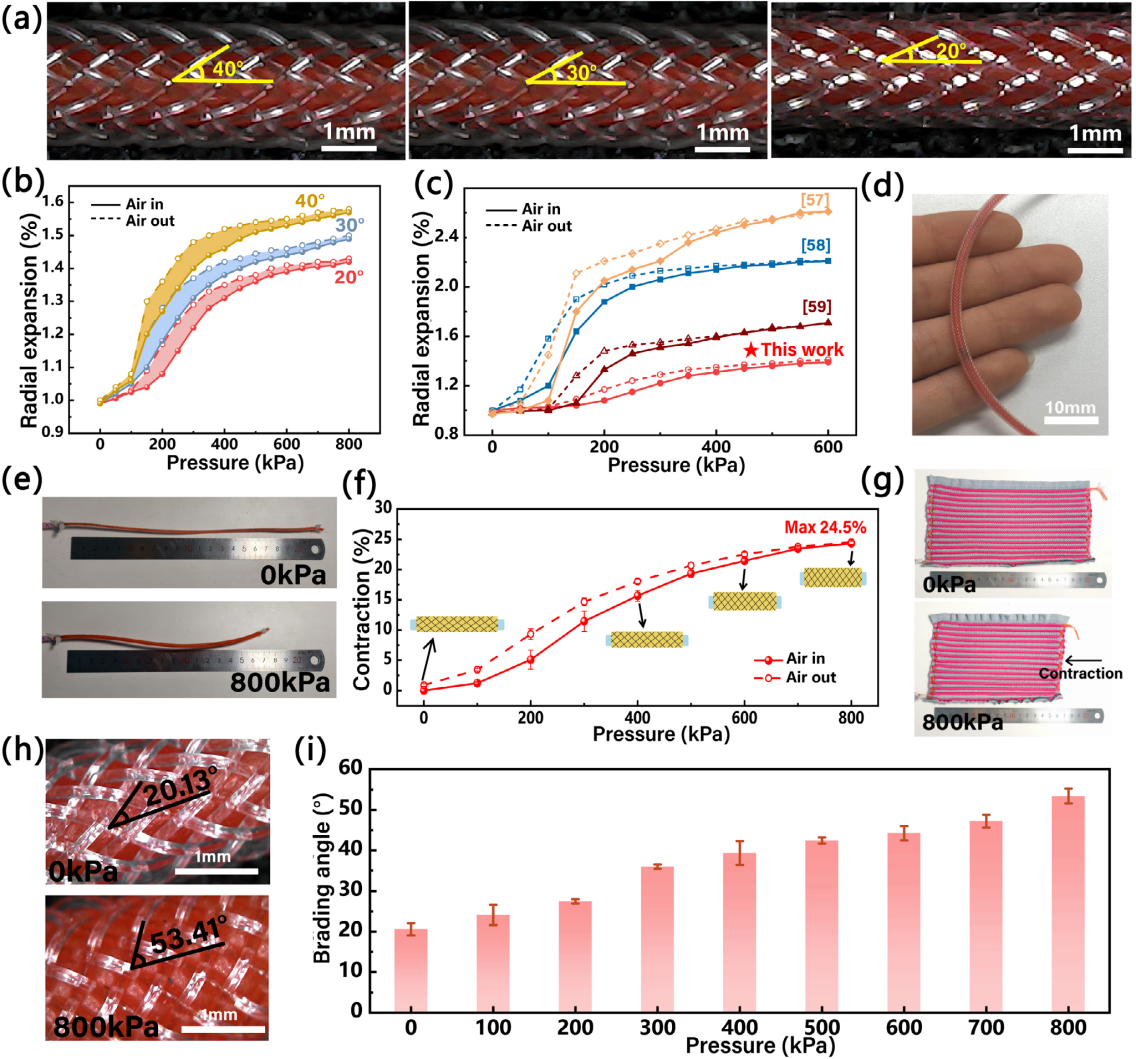
The characterization of the pneumatic fibers. (a) Schematic and electron micrograph images (scale bar: 400 µm) of the pneumatic fiber with braiding angles of 40^°^, 30° and 20°. (b) Radial expansion of the pneumatic fiber with braiding angles of 40°, 30° and 20° under pressure from 0 to 800 kPa. (c) Comparison of the expansion rate of the pneumatic fiber with currently reported PAMs [57, 58, 59]. (d) Braiding angle of 20^°^ in the pneumatic fiber. (e) Contraction of the pneumatic fiber with a 20° braiding angle at 0 and 800 kPa). (f) Contraction characterization of the pneumatic fibers with a 20° braiding angle. (g) The contraction of the PPKF. (h) Electron micrograph images (scale bar: 400 µm) of the PPKF at 0 and 800 kPa. (i) Variation of the braiding angle of the pneumatic fiber under pressure from 0 to 800 kPa.

Considering the wearability, the radial expansion of the pneumatic fiber under pressure significantly affects the PPKF knitting process, driving volume, and deformation stability. Moreover, the textile's extensibility, compressibility, and breathability critically determine the wearability of garment [54]. The braiding angle of outer mesh is an important factor affecting the properties of the pneumatic fiber [42]. Since braiding angles <54.4° induce contraction of the pneumatic fiber but must exceed 18° [43]. On this basis, in order to achieve low radial expansion of the pneumatic fiber, we adjusted the braiding angle (20°, 30° and 40°, Figure 3a) by changing the yarn tension and yarn placement angle during the braiding process. Comparative analysis of three braiding angle of the pneumatic fibers at 0–800 kPa, shown in Figure 3b. The maximum radial expansion rate of the pneumatic fiber with 40° braiding angle is 1.58%, while 20° braiding angle is only 1.41%. Comparative analysis demonstrates that these pneumatic fibers significantly outperform most reported PAMs (Figure 3c; Table S1). These results demonstrate reduced braiding angles in the pneumatic fibers correlate with lower radial expansion rates. Consequently, we integrated the pneumatic fiber with 20° braiding angle (Figure 3d) into the PPKF structure for subsequent research. This performance advantage stems from the pneumatic fiber's core‐sheath configuration: an elastomeric bladder tightly coupled with an external braided mesh. The resulting core‐sheath assembly enables uniform contractile deformation, enhancing PPKF integration within knitted architectures while facilitating compression garment miniaturization and improving wearability.

Notably, 20° braiding angle of the pneumatic fiber achieves maximum 24.5% contraction under 0 – 800 kPa(Figure 3e,f). This closely matches the contraction range of human muscle fibers (20–30%) [55], which enhances the fabric's fit to the body. By embedding pneumatic fibers into knitting structure, the PPKF gains the capability to contract. (Figure 3g). Concurrently, the braiding angle of the pneumatic fiber increases from 20.13° to 53.4°under equivalent pressures (Figure 3h,i; Movie S2), aligning with the maximum angle (54.4°) reported for most PAM [56] and proving the effectiveness of the pneumatic fiber. The data reveal that the braiding angle of the pneumatic fiber increases rapidly at 200–300 kPa. This is because when the input pressure is less than 300 kPa, there is a certain distance between the inner bladder and the external braid mesh. When the outer wall of the inner bladder comes into full contact with the braid mesh at around 300 kPa, the braiding angle increases rapidly due to the interaction of forces, causing the pneumatic fiber to contract significantly.

**FIGURE 4:**
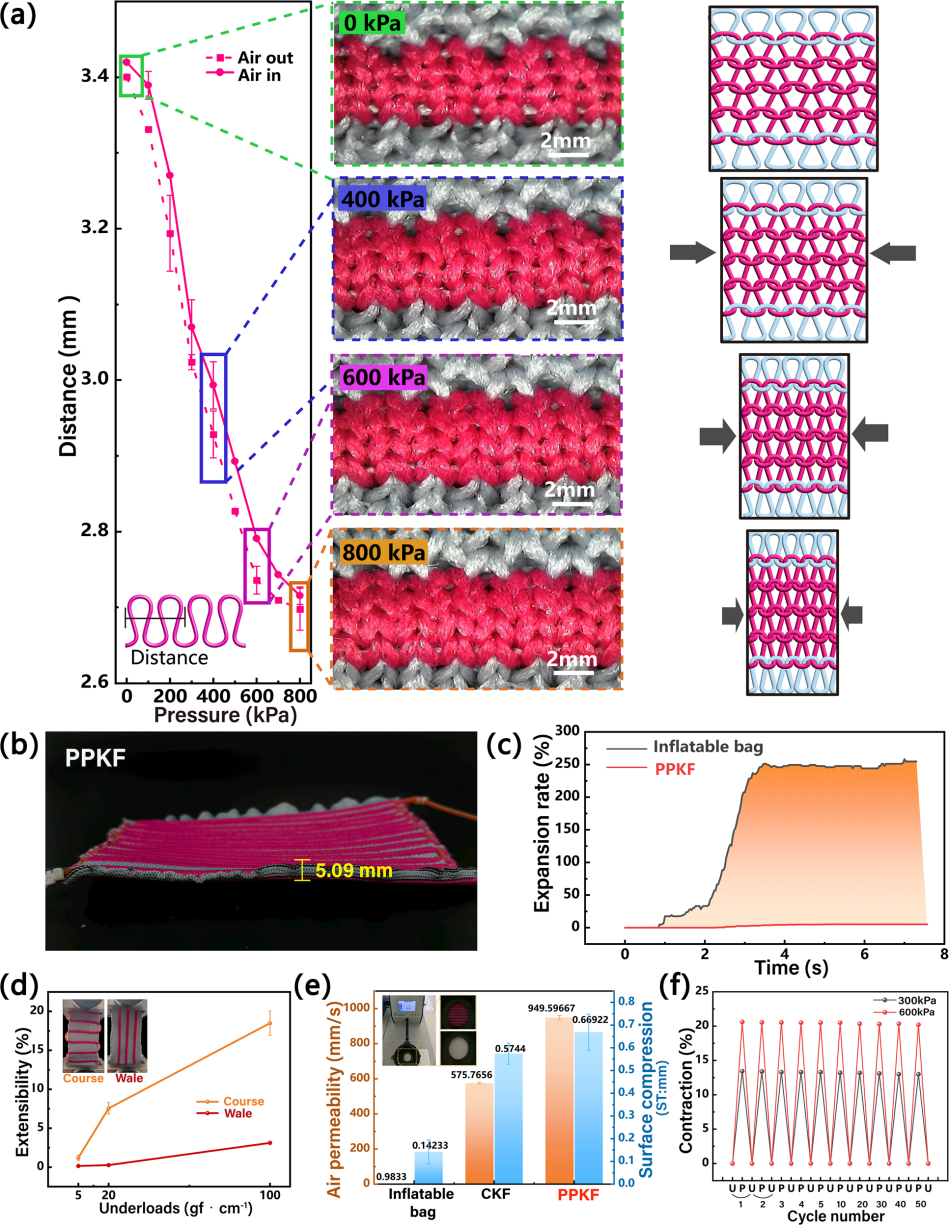
Wearable performance of the PPKF. (a) Distance between adjacent knitted loops and schematic of loop deformation at 0–800 kPa. (b) The radial expansion of the PPKF is shown to be a maximum of 5.09 mm. (c) Radial expansion rate of inflatable bag fabrics and the PPKF at the same inflation time. (d) Characterization of the PPKF extensibility in the course and wale directions. (e) Comparison of air permeability and surface compression among inflatable bag fabrics of equivalent thickness, compression knit fabrics (CKF) and the PPKF. (f) Reversible cyclic variation of the PPKF contraction over 50 cycles at different pressures (300 and 600 kPa).

To quantify the structural changes in the knitted fabric induced by the pneumatic fiber, we measured the distance between two adjacent knitted loops at 0–800 kPa (Figure 4a). In its initial state (0 kPa), the PPKF features loose knitted loops. Upon the application of increasing air pressure, the pneumatic fibers contract progressively. This action gradually tightens the knitted loop spacing, resulting in the overall contraction of the fabric. Quantification comparison of radial expansions is shown in Figure 4b,c. Impressively, the PPKF exhibits minimal radial expansion (max 5.2%, diameter increase to 5.09 mm) across 0–800 kPa (Figure 4c). This ultralow radial expansion effectively stabilizes minimizes fabric volume during actuation. In contrast, conventional sealed‐chamber inflatable bag show dramatic radial expansion (> 250.0%, Movie S3), which is 48‐fold greater than PPKF. This substantial expansion leads to poor fit of complex body contours.

PPKF avoids these limitations by restricting radial expansion through two critical mechanisms: the pneumatic fiber's ultralow‐expansion properties and its tight matrix integration. This embedded configuration enables synergistic interaction between the braided fiber network and knitted substrate, collectively minimizing deformation under pressurization. The quantitative comparison of wearability is shown in Figure 4d. Under applied loads (20–100 gf·cm^−1^), the PPKF exhibits greater extensibility in the course direction (max 18.49%) versus the wale direction. This directional anisotropy enables the fabric to accommodate lateral body dimension changes, fit to body curves, and reduce pressurization‐induced tightness. It is worth noting that the lower extensibility of the PPKF in the wale direction is primarily due to the constraining influence of the pneumatic fiber (comprising the elastic inner bladder and relatively stiff nylon monofilament).

As shown in Figure 4e, the PPKF exhibits exceptional air permeability (949.6 mm/s) and compressibility (0.669 mm). These properties are crucial for addressing breathability and fit issues in pneumatic fabrics. In comparison, compressed knitted fabrics (CKF) of equivalent thickness show inferior performance (air permeability: 575.8 mm/s; compressibility: 0.574 mm), while traditional inflatable bag fabrics perform significantly worse (air permeability: 0.983 mm/s; compressibility: 0.142 mm). The superior air permeability and compressibility of the PPKF stem from its unique loop structure, which enables loop deformation and slippage under pressure. Importantly, the pneumatic fibers are embedded within dual‐layer channels rather than enclosed spaces, while inherent inter‐loop pores enhance air permeability. Conversely, most compression fabrics employ tight weaving for pressure requirements, and inflatable bag materials utilize non‐breathable layers to maintain chamber integrity, both compromising breathability. Consequently, the PPKF is ideal for compression garments requiring high compressibility, extensibility, and breathability. Furthermore, the good repeatability and durability of the PPKF are proved by the stable contraction test over 50 cycles (Figure 4f), which indicates robust performance of the PPKF and can be used for practical applications.

### 2.3 Mechanisms and Design of Dynamic Modulating Gradient Pressurization

**FIGURE 5:**
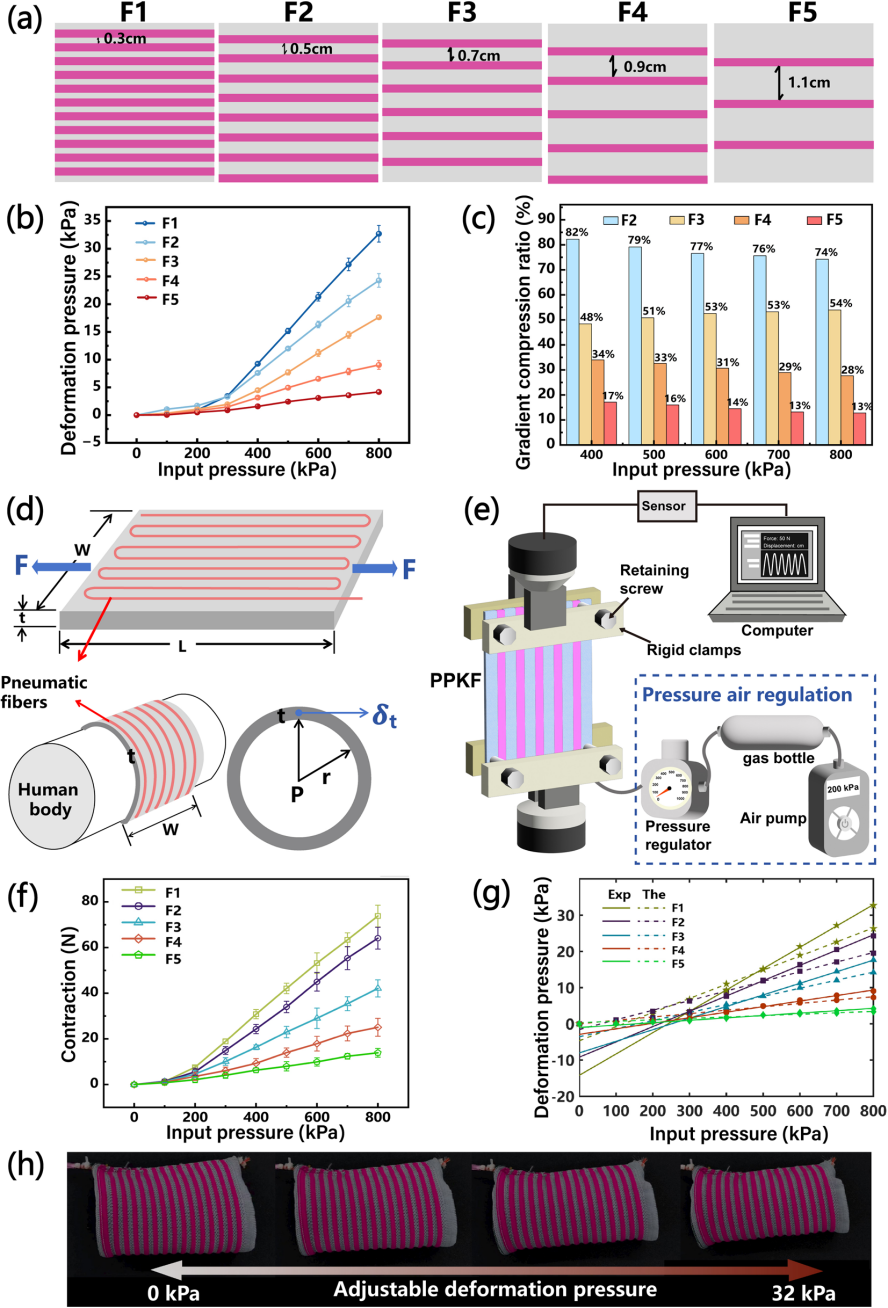
Mechanisms and design of dynamic modulating gradient pressurization. (a) Schematic of five PPKFs (F1–F5) with varying channel distance parameters. (b) Experimental deformation pressure data for F1–F5 (0–800 kPa). (c) Calculated gradient compression ratios derived from the data in (b). (d) Schematic illustrating the theoretical model parameters for PPKF deformation pressure. (e) The contraction testing of PPKF. (f) Results of compression force tests for five PPKFs. (g) The linear fitting results of the theoretical deformation pressure calculated from (f) and experimental data. (h) Sequence image from Movie S4 showing adjustable deformation pressure process of a PPKF at 0–800 kPa.

The PPKF can be manufactured on knitting machines. A 3D tube structure is formed by linking both edges with elastic yarns using a linking machine, which minimizes constraints between loops and maximizes fabric elongation (details in Experimental Section and Figure S5). Further theoretical analyses were conducted. First, we investigated the mechanism of controllable compression generation in PPKF (Figure S6). Dynamic modulating gradient pressurization is achieved by modulating channel spacing *d*. Parameterizing this spacing, five PPKF samples (F1‐F5) were fabricated with the distance *d* (0.3, 0.5, 0.7, 0.9, and 1.1 cm) between two adjacent channels (Figure 5a; Figure S7). The deformation pressures of these PPKFs with different channel densities were measured across 0–800 kPa following the methodology of Granberry et al. [60]. (Figure S8).

As shown in Figure 5b, experimental results indicate that the PPKF deformation pressure scales with input pressure. Crucially, reduced channel spacing *d* yields higher deformation pressure. At 800 kPa, F1 (*d* = 0.3 cm) generated maximum deformation pressure (32.7 kPa), while F5 (*d* = 1.1 cm) produced minimum pressure (4.18 kPa). Notably, above 300 kPa, all samples exhibited accelerated linear pressure increase. This trend aligns with prior pneumatic fiber characterization and indicates a stabilization threshold at 300 kPa, confirming precise deformation pressure controllability.

Given these findings, deformation pressure data within the 400–800 kPa range were analyzed for gradient compression ratio. F1 (generating maximum pressure) served as the 100% reference baseline. Gradient compression ratios for F2‐F5 were calculated relative to F1, demonstrating consistent gradient compression profiles (Figure 5c). The final compression ratios: 100.0% (F1), 77.6% (F2), 51.8% (F3), 31.0% (F4) and 14.6% (F5), closely approximate medical gradient compression standards [22].

Second, a theoretical model based on Laplace's Law was developed to analyze the compression mechanism of the PPKF. Assuming that the human body can be approximated as a uniform thin‐walled cylinder of radius *r*, the PPKF can be modeled as a cylindrical hollow thin‐walled container with thickness *t*, width *W*, and length *L*. Upon pressurization and contraction, the PPKF exerts a circumferential contraction force *F* (Figure 5d). Therefore, the transversal stress σ_t_ is given by:

(1) $$\sigma_{t}=\frac{F}{tW}\;$$

Transverse stresses are generated by the pressure *P* in the inner cavity of the PPKF:

(2) $$\sigma_{t}=\frac{Pr}{t}\;$$

From formulas (1) and (2), we obtain:

(3) $$\frac{F}{tW}=\frac{Pr}{t}\;$$

(4) $$P=\frac{F}{\mathrm{rW}}\;$$

This results in a final PPKF deformation pressure of:

(5) $$\mathrm{Deformation}\;\mathrm{pressure}\propto \frac{F}{rW}$$

Five samples were experimentally characterized following Equation (4)’s theoretical model. Contraction forces generated by PPKF across 0–800 kPa input pressure were measured via Kurumaya's methodology [57]. The contraction testing is shown in Figure 5e, more details shown in Figure S9. The PPKF contraction force increased with input pressure, mirroring the deformation pressure trend, and peaked at 73.8 N (800 kPa, Figure 5f). The contraction force data were applied to Equation (4) to calculate theoretical deformation pressure. Linear regression analysis revealed near‐perfect agreement between theoretical and experimental values (R^2^> 0.995). Below 300 kPa, the pneumatic fiber pressure had not stabilized, resulting in non‐linear force contraction growth. Above this threshold, both theoretical predictions and experimental deformation pressure exhibited linear growth (Figure 5g), demonstrating controllable PPKF deformation beyond the stabilization point. Interestingly, theoretical‐experimental correlation improved with larger channel spacing *d*, as increased spacing reduces the pneumatic fiber density in the textile matrix, minimizing deformation pressure variance across pressure gradients. Furthermore, contraction force measurement requires pressurized fixture displacement, inevitably inducing air leakage, particularly at higher pressures, resulting in values below theoretical expectations and introducing error into linear model fitting. Critically, results confirm deformation pressure proportionality to $\frac{F}{rW}$, with experimental proportionality constant *k*. The PPKF enables controlled compression by adjusting the channel spacing without complex segmentation (Figure 5h; Movie S4), while its inherent conformability adapts to irregular body shapes for precise medical gradient compression.

Additionally, we integrated a soft sensor feedback (Elastreme Sense Slim) for real‐time pressure feedback, demonstrating stable voltage signals during cyclic loading (Figure S10). This implementation reveals a promising pathway for embedding textile‐based stress sensors within pneumatic wearables to enable real‐time deformation monitoring. Critically, our integrated manufacturing strategy offers simplicity, scalability, and cost efficiency, distinguishing it from conventional multi‐component pneumatic systems (Figure S11 and Table S2).

### 2.4 Applications for Healthcare Wearables

**FIGURE 6:**
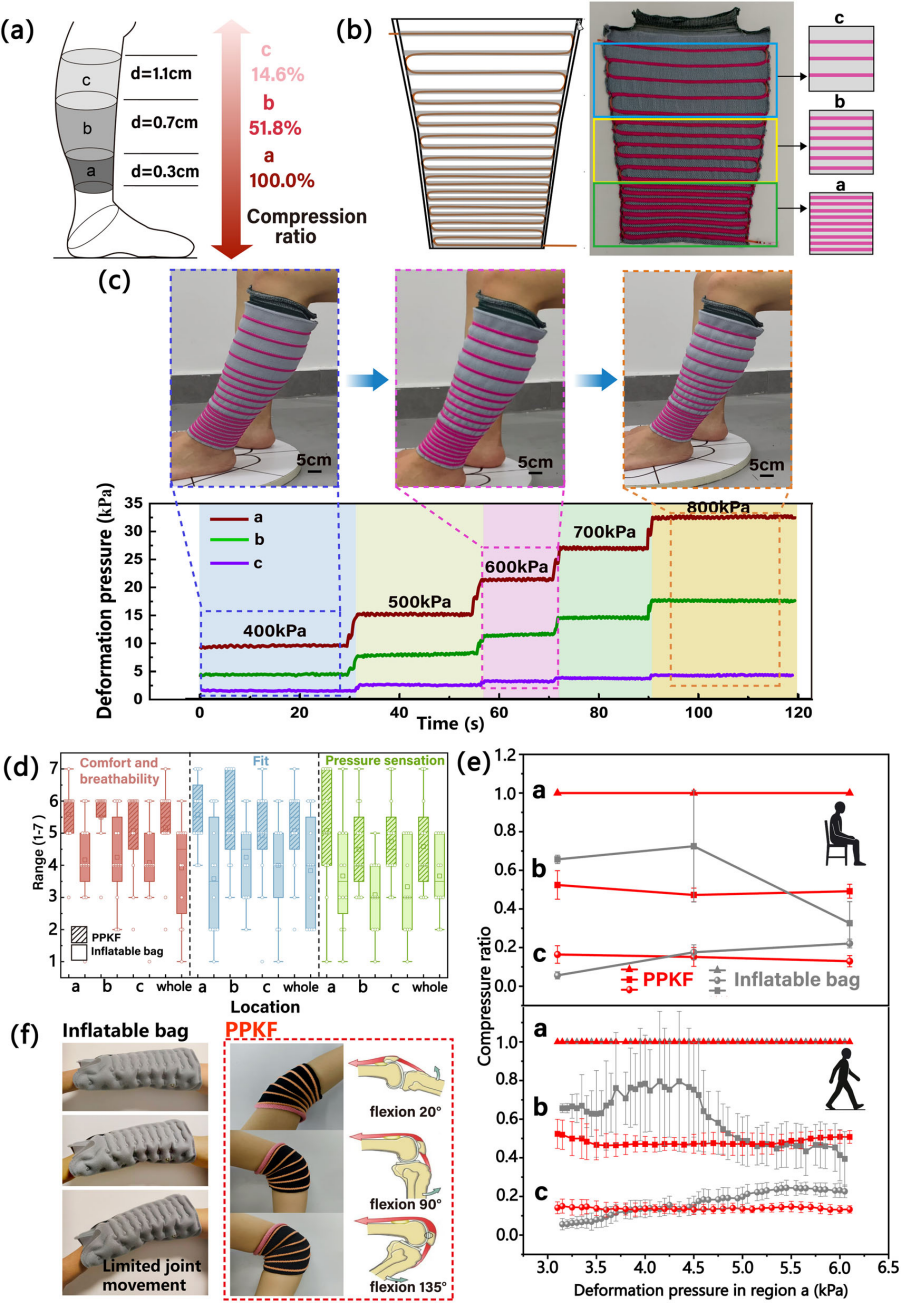
Application of the PPKF in medical gradient compression garments. (a) Division of lower limb from a to c into three distinct compression zones based on predefined gradient compression ratios (Section 2.3) and PPKF channel spacing. (b) Knitting pattern for the pneumatic gradient compression calf sleeve produced using a flat knitting machine. (c) Stable deformation pressure output (400–800 kPa) with simultaneous gradient pressurization across all three zones. (d) The results of subjective rating evaluation of the compression calf sleeve and inflatable bag (n = 12). (e) The results of objective pressure test in sit and walk (4 km/h). (f) Application of PPKF in joint areas.

Unlike conventional pneumatic therapy textiles, PPKF employs miniaturized embedded pneumatic fibers. This innovation drastically reduces pneumatic volume requirements, enabling air storage in compact gas bottle. Consequently, ancillary hardware (gas supply and pressure regulation) is miniaturized to smartphone‐scale dimensions (Figure S12), eliminating bulky external pumps and enhancing portability. We fabricate gradient compression calf sleeves via direct knitting for venous insufficiency therapy (Figure 6a,b). The gradient compression ratio was set according to established values (Section 2.3). Programmable stitch modulation during knitting enables precise anatomical conformity to lower extremity curvature (Figure S13), ensuring optimal fit and enhancing therapeutic pressure distribution accuracy. Unlike conventional pneumatic garments requiring segmented inflatable bag with multiple pneumatic interfaces, our knitted calf sleeve achieves zoned compression regulation structurally using just a single pneumatic interface. This integration streamlines manufacturing while enhancing efficiency. As fabricated (Figure 6b), the sleeve provides gradient compression across three lower leg zones: a (100.0%), b (51.8%), c (16.4%). This structural design enables effective pressure gradient control, delivering 5–32 kPa compression at 400–800 kPa inflation (Figure 6c). The favorable compression gradient across zones a–c enables straightforward regulation in practical applications.

To validate the wearability of PPKF, we conducted subjective rating evaluation and objective pressure test (Figure S14). A commercially pneumatic compression garment (yht21A, Ningbo Haorou Medical Technology Co., Ltd., China) was selected as the reference garment and made of inflatable bag. Subjective rating evaluation involved 12 participants. Simultaneously evaluate the overall garment and the three key areas of the lower limbs. Compared to inflatable bag, the pneumatic gradient compression calf sleeve that was made of PPKF showed greater comfort and breathability, fit and lower constriction in a–c areas and the whole garment. The results showed that the PPKF had mean scores of 5/7 for comfort and breathability, fit of whole garment, about 4.58/7 for pressure sensation (Figure 6d). In objective test, compared to inflatable bag, we observed that gradient compression garments manufactured by PPKF exhibited more stable gradient compression ratio under both static (sitting) and dynamic (4 km/h walking) conditions (Figure 6e). Combined subjective and objective evaluation, the results indicated better wearability for the developed pneumatic gradient compression calf sleeve.

Furthermore, this technology shows significant potential for dynamically pressurized wearables. The knitting structure can be readily adapted based on the direction of skin stretch at high‐mobility areas, like joints, allowing the garment to conform to joint movement. For compression garments designed to operate around human joints, excessive radial expansion can be detrimental to mobility. And we further demonstrate the application of the PPKF at articulated joint regions (Figure 6f). Compared to conventional inflatable bag, which rely on substantial radial expansion to generate pressure, the PPKF maintains effective axial compression while substantially suppressing radial deformation, thereby adapting to body contours. When applied to joint areas, such as the knee or elbow, this low radial expansion prevents local bulging and avoids mechanical interference with joint bending. Additionally, garment fit can be improved by adjusting knitting parameters. As a result, the garment is able to conform to joint motion and maintain consistent compression during dynamic movements. In contrast, traditional inflatable bag structures undergo pronounced radial expansion under pressurization, leading to increased local thickness around the joint, which restricts flexion and hinders natural movement.

This capability is essential for developing dynamically gradient‐pressured pneumatic knitted garments, potentially overcoming mobility limitations in conventional pneumatic garments and enabling next‐generation dynamically pressurized systems.

## 3 Conclusion

To advance functional healthcare wearables, this study proposes a dynamic modulating Pneumatic Pressure‐Regulating Knitted Fabric (PPKF) system based on biomimetic fiber and knitting structure. The validity of the approach was verified through comparative analysis with existing pneumatic artificial muscles (PAMs) and medical standards. The PPKF enhances pressure‐regulation precision and fit to body while improving wearability, enabling novel therapeutic garment design. The main conclusions are as follows:

1. A pneumatic pressure‐regulation platform was established using integral knitting technology, with performance validated against medical standards. The PPKF achieves ultralow radial expansion (≤ 5.2%) and biomimetic contraction (24.5% at 800 kPa), demonstrating an expansion rate up to 48 times lower than that of conventional inflatable bag, while still covering the full range of necessary medical compression ratios (14.6 to 100.0%).
2. Gradient pressurization is dynamically controllable through channel‐spacing modulation (0.3–1.1 cm), enabling adjustable pressure output (3–32 kPa). The system maintains stability above 300 kPa, with theoretical models showing strong agreement with experimental data (R^2^ > 0.995).
3. Integrated manufacturing enables body‐conforming pneumatic textiles with superior breathability (949.6 mm/s) and fit the body shape. Subjective evaluations confirm comfort and breathability, fit (> 5/7), minimal constriction sensation (about 4.58/7). Objective testing demonstrates that PPKF produces more stable gradient compression in both static and dynamic conditions.

The PPKF can be extended to diverse medical and athletic wearables requiring dynamic compression. It serves as foundational technology for next‐generation therapeutic garments while reducing system complexity through single‐interface operation. However, it should be clearly noted that current validation focuses on lower limbs of the human body. Future research will explore the adaptability to other body parts, such as soft gloves, and long‐term clinical efficacy. In addition, due to the smaller fiber diameter and the weakness in radial expansion, it consumes more pressure. Future work will focus on reducing the required operating pressure through several complementary strategies.

## 4 Experiment

### 4.1 Materials

The elastomeric bladder was purchased from Shanghai Gukai New Material Technology Co (ID: 1 mm, OD: 2 mm). Nylon monofilament (0.2 mm diameter) was purchased from a company (Zhejiang Hengting Emperor Co., Ltd.). High elasticity yarn (100 d) and low elasticity yarn (100d) were purchased from Ningbo Cixing Co. Elastreme Sense Slim was purchased from Beijing Rouzhi Technology Co., Ltd.

### 4.2 Braiding of the Pneumatic Fiber

A pneumatic fiber was braided using a braiding machine (Model 140‐24, Xuzhou Qixing Machinery Co., Ltd., China). An elastomeric bladder was placed in the center of the braiding area. The machine's spool rotated clockwise, wrapping the nylon monofilament around the bladder. The braiding machine's operating parameters (spindle speed set to 8000 rpm) were adjusted to maintain a constant braiding angle to produce the pneumatic fiber. The relative humidity was maintained above 20 ± 2% and above 65 ± 2% during the braiding process (Details in the Supporting Information).

### 4.3 Fabrication of the PPKF

The PPKF was fabricated on a two‐needle‐bar knitting machine (STG3.132MC‐U‐1‐14G, Ningbo Cixing Co., Ltd., China) with a gauge of 14 and knit width of 132 cm (52 inch). Three strands of 100 d high‐elastic yarn and low‐elastic yarn were used for the dual‐layer hollow channel and single‐layer structures of the PPKF, respectively. The boucle yarns were hooked by the front and back knitting needles at intervals to interlace with each other to alternatively form the front and back loops. The pneumatic fiber is feed into customized large aperture yarn feeders (Figure S4). The pneumatic fiber was knitted as weft inserts, without forming loops during the knitting process. The pneumatic fiber was embedded between loop pairs with the needle position of 0.00P.

### 4.4 Fabrication of the 3D tube Knitting Fabric

The PPKF was woven into a 200 mm × 100 mm rectangular shape and the sample was sewn into a 3D tube using high elastic yarn. A linking machine (GD‐A200L, Dongguan Fengshen Machinery Co., Ltd., China) was chosen for sewing because it minimizes restrictions between knitted loops and maintains the elasticity of the knitted fabric (Figure S5). One end of the pneumatic fiber embedded in the PPKF was connected to an inflation connector and the other end was tied off with cable ties to avoid any possible air leakage.

### 4.5 Characterization

A XL‐1A (20) Yarn Tensile Tester (Shanghai Xinxian Instrument Co., Ltd., China) was used to measure the tensile properties of yarn and elastomeric bladder with a gauge of 100 mm and testing speed of 200 mm min^−1^. The changes in the braiding angles of the pneumatic fiber and the distance of knitted loop was measured using an electron microscope (su 8010, SEM, China). In the tensile measurements, samples were clamped at a distance of 100 mm and stretched at a test speed of 100 mm min^−1^. A digital thickness gauge (Everte, China) was used to measure the diameter of the pneumatic fiber at different air pressures. The expansion ratio is expressed as:

(6) $$\mathrm{Radial}\;\mathrm{expansion}\;\left(\%\right)=\frac{\Delta D}{D_{0}}\;$$

where Δ*D* is the difference between the fiber diameter at different air pressures and the initial fiber diameter *D_0_*. A high‐speed camera (Micro LAB‐110, Ametek, USA) was used to record the expansion rate of the fabric after inflation deformation. Tracker software was used to quantify fabric expansion. The Fabric Assurance Simple Test (FAST, UK) system was used to characterize the surface compression and extensibility of fabrics. Compression test: flat fabric samples (at least 100 × 100 mm) were tested under pressures of 2 and 100 gf/cm^2^ (*n* = 5). Surface thickness (*ST*: mm) was computed as the difference in thickness under low and high pressure.

(7) $$\mathrm{ST}=T_{2}-T_{100}$$

where *T_2_* and *T_100_* are the average thickness for a load of 2 and 100 gf/cm^2^, respectively.

Extensibility test: The PPKF was woven into 50 mm × 130 mm rectangular shape in the warp and weft directions. The warp and weft extensibility were measured under loads of 5, 20, and 100 gf·cm^−1^. According to the China standard of GB/T 5453‐1997 Textiles‐Determination of the permeability of fabrics to air, the air permeability of the fabrics was tested on a YG461G automatic air permeability measuring instrument, setting the test pressure to 100 Pa and the test area of the samples to be 20 cm^2^. The results were obtained by averaging the values of three specimens.

Deformation pressure test: a Novel Pliance pressure mapping system was used to test the deformation pressure of the PPKF. Pressure sensors positioned at three circumferential locations on the 3D tube structure recorded pressures from 0 to 800 kPa.

Contraction force test: referring to the method of Hiramitsu et al. [45]. An electronic universal tester (Instron 3400, USA) was used to test the contraction force of the PPKF under internal pressure (Figure S9). Both ends of the PPKF were secured with rigid clamps (200 mm × 40 mm) and screws to prevent slippage. After applying 5 N of pre‐tension and resetting the force sensor to zero, the corresponding air pressure was input, and the sensor force value *F* was recorded. Considering that the clamp will generate a certain force, the clamps were displaced along the PPKF's contraction direction until the force sensor registered 0 N. The clamp was pulled back to the initial length of the sample, and the sensor force value *F'* was recorded, which is the contraction force of the PPKF. Triplicate measurements were averaged for final analysis. (Details in the Supporting Information).

### 4.6 Objective Pressure Test and Subjective Rating Evaluation

In objective pressure test, a Novel Pliance pressure mapping system was used to test the deformation pressure of the gradient compression garments. A commercially pneumatic compression garment (yht21A, Ningbo Haorou Medical Technology Co., Ltd., China) was selected as the reference garment and made of inflatable bag. Reference to RAL‐GZ 387 [5], sensors are placed at three key regions on the lower limb (a,b, and c), close to the great saphenous vein (Figure S14). Applying pressure of 3.1–6.1 kPa (23–46 mmHg) to the ankle (region a), which is the commonly used pressure for treating varicose veins. The final result is calculated based on the compression ratio. Garment pressures were recorded at three predefined lower‐limb regions (*P_a_*, *P_b_*, and *P_c_*), regional pressures were normalized to ankle pressure *P_a_*. The resulting compression ratios for the three regions were defined as *R_a_*, *R_b_*, and *R_c_*, and calculated as follows:

(8) $$R_{a}=\frac{P_{a}}{P_{a}}\;,\;R_{b}=\frac{P_{b}}{P_{a}}\;,\;R_{c}=\frac{P_{c}}{P_{a}}\;$$

In subjective rating evaluation, the wearable evaluators included 12 participants aged 24–35 years. Written informed consent was obtained from all participants before data collection. The study protocol was reviewed and approved by the Ethics Committee of the College of Fashion and Art Design, Donghua University (No: RLSSZYJ202511150078), and was conducted in accordance with relevant ethical guidelines for human‐subject research. Subjective evaluation areas were conducted for each of the three lower leg areas (a,b, and c) and the overall garment (whole), while wearing both garments. Participants’ self‐assessments were standardized on a 7‐point scale [61, 62], and they rated the comfort and breathability, fit, and pressure sensation of the garments after trying the pneumatic gradient compression calf sleeve and inflatable bag. The scale anchors were defined as follows:

·Comfort and breathability: 1 = very uncomfortable, 7 = very comfortable

·Fit: 1 = poor fit, 7 = excellent fit

·Pressure sensation: 1 = strong pressure, 7 = mild pressure

### 4.7 Statistical Analysis

The quantitative data with error bar are represented by mean ± S.D. based on at least three replicates unless stated otherwise. Statistical analysis was conducted on Origin 2018 or Microsoft Excel 2019.

## Funding

National Natural Science Foundation of China (Grant No. 52576063)

## Conflicts of Interest

The authors declare no conflicts of interest.

## Supporting information




**Supporting File 1**: advs74688‐sup‐0005‐SuppMat.docx.

**Supporting File 2**: advs74688‐sup‐0001‐MovieS1.mp4.

**Supporting File 3**: advs74688‐sup‐0002‐MovieS2.mp4.

**Supporting File 4**: advs74688‐sup‐0003‐MovieS3.mp4.

**Supporting File 5**: advs74688‐sup‐0004‐MovieS4.mp4.

## Data Availability

The data that support the findings of this study are available in the supplementary material of this article.
