# Supplementary material for: Knitted Pneumatic Fabrics for Dynamic Pressure Modulation in Personalized Healthcare Wearables
Source: Adv Sci (Weinh). 2026 Mar 27;13(27):e18305. doi: 10.1002/advs.202518305 (PMC13170273; doi:10.1002/advs.202518305)
Supplement: Supplementary file 1 — Supporting File 1: advs74688‐sup‐0005‐SuppMat.docx. [file ADVS-13-e18305-s001.docx]

Supporting Information for

Knitted Pneumatic Fabrics for Dynamic Pressure Modulation in Personalized Healthcare Wearables

Xiaoyu Chen ^a^, Jintu Fan ^b, c, d, *^, Rong Zheng ^d, *^, Qing Chen ^d^, Fengxin Sun ^e *^, Huanhuan Liua ^a^

a. College of Fashion and Design, Donghua University, Changning District, Shanghai 200050, China

b. School of Fashion and Textiles, The Hong Kong Polytechnic University, Hung Hom, Kowloon, Hong Kong 999077, China

c. PolyU Xingguo Technology and Innovation Research Institute, Research Centre of Textiles for Future Fashion and School of Fashion and Textiles, The Hong Kong Polytechnic University, Hong Kong 999077, China

d. Shanghai International Fashion Innovation Center, Donghua University, Shanghai 200050, China

e. Laboratory of Soft Fibrous Materials & Physics, Jiangnan University, Wuxi 214122, China

* Corresponding authors: rzheng@dhu.edu.cn (R. Zheng), jin-tu.fan@polyu.edu.hk (J. Fan),

[fxsun@jiangnan.edu.cn](mailto:fxsun@jiangnan.edu.cn) (F. Sun)

**This file includes:**

Supporting Figures S1 to S13

Figure S1 Traditional inflatable bag textiles.

Figure S2 The manufacturing and details of the pneumatic fiber.

Figure S3 The mechanical properties of PET yarn and elastomeric bladder.

Figure S4 The textile production processes for the PPKF.

Figure S5 Fabrication of a 3D PPK.

Figure S6 Mechanism of controllable compression in PPKF.

Figure S7 Five PPKF samples with different channel densities.

Figure S8 Deformation pressure test.

Figure S9 Contraction test.

Figure S10 Real-time pressure feedback based on soft sensors (Elastreme sense slim).

Figure S11 A comparative analysis of the PPKF with the main performance characteristics of current compression garments.

Figure S12 Wearable air supply equipment.

Figure S13 The gradient compression ratio and knitting pattern of the pneumatic gradient compression calf sleeve.

Figure S14 Objective pressure test and subjective rating evaluation.

Supporting Table S1 to S2

Table S1 Comparisons of PAM in current research

Table S2 Comparisons of reported compression garments by ordinal numbering

**Other supplementary materials for this manuscript include the following:**

Movies S1 to S4

Movie S1: Contractible deformation of the pneumatic fiber.

Movie S2: The change in the braiding angle of the pneumatic fiber at 0 - 800 kPa.

Movie S3: The radial expansion of the inflatable bag and PPKF.

Movie S4: Contractible deformation of PPKF

**References for Supporting Information**

**Supporting Figures (Figure S1-S14)**


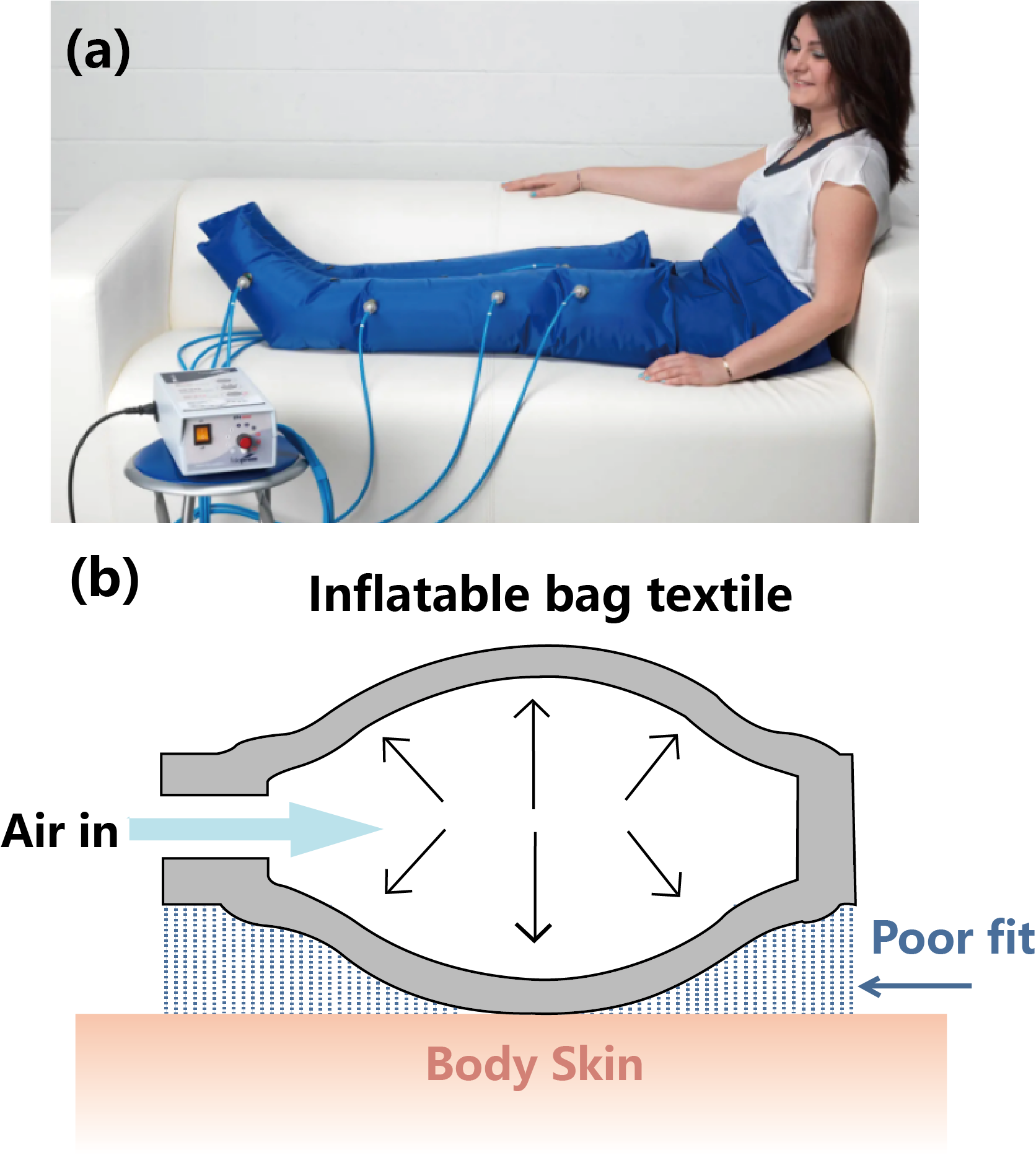


**Figure S1.** Traditional inflatable bag textiles. (a) Commercial medical compression garments — Fisiopress.^[1]^ (b) The radial expansion of the inflatable bag textile causes uneven pressure and poor fit.

**
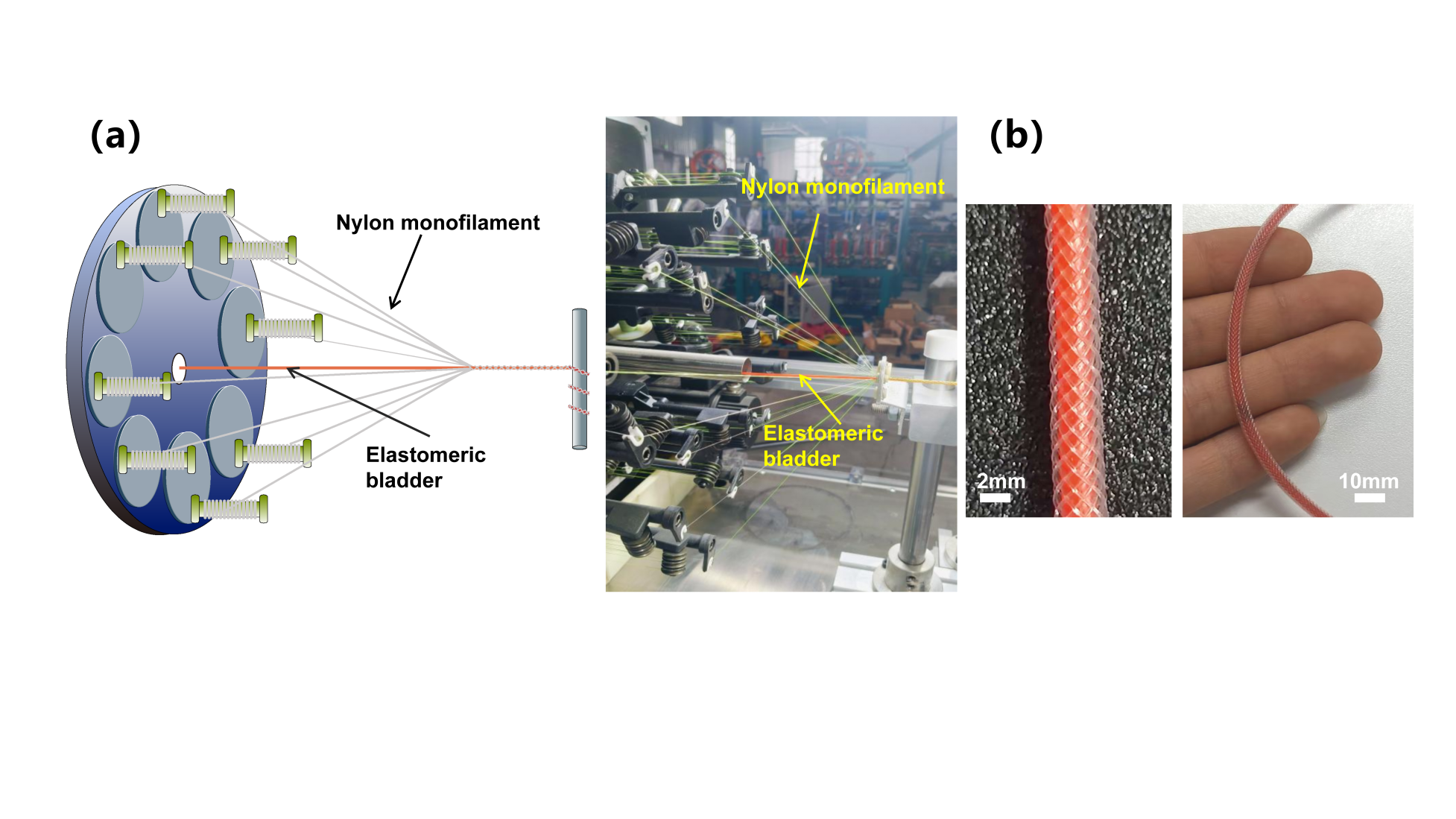

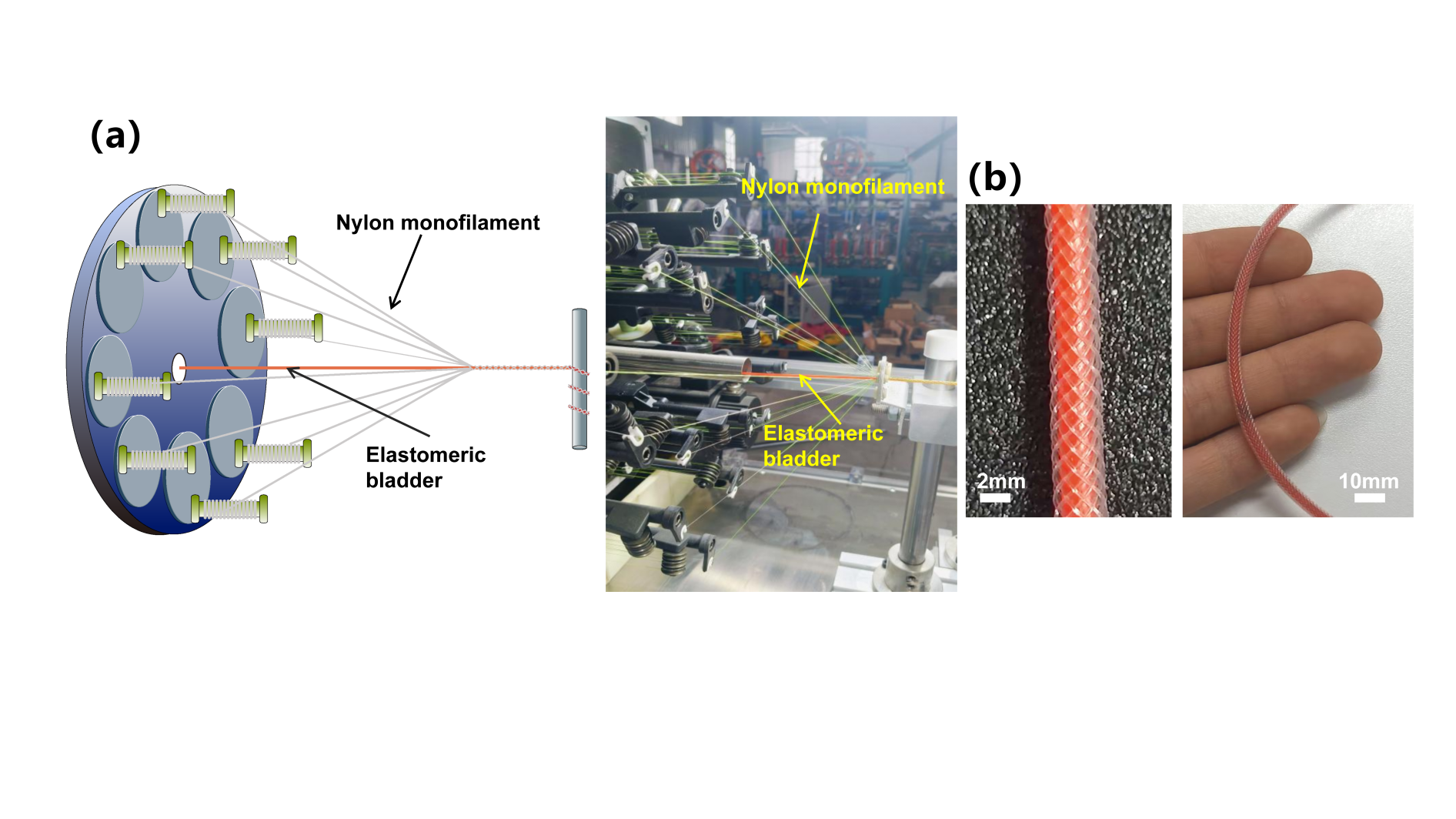
**

**Figure S2.** The manufacturing and details of the pneumatic fiber. (a) Integrated manufacturing of the pneumatic fiber. (b) The details of the pneumatic fiber.


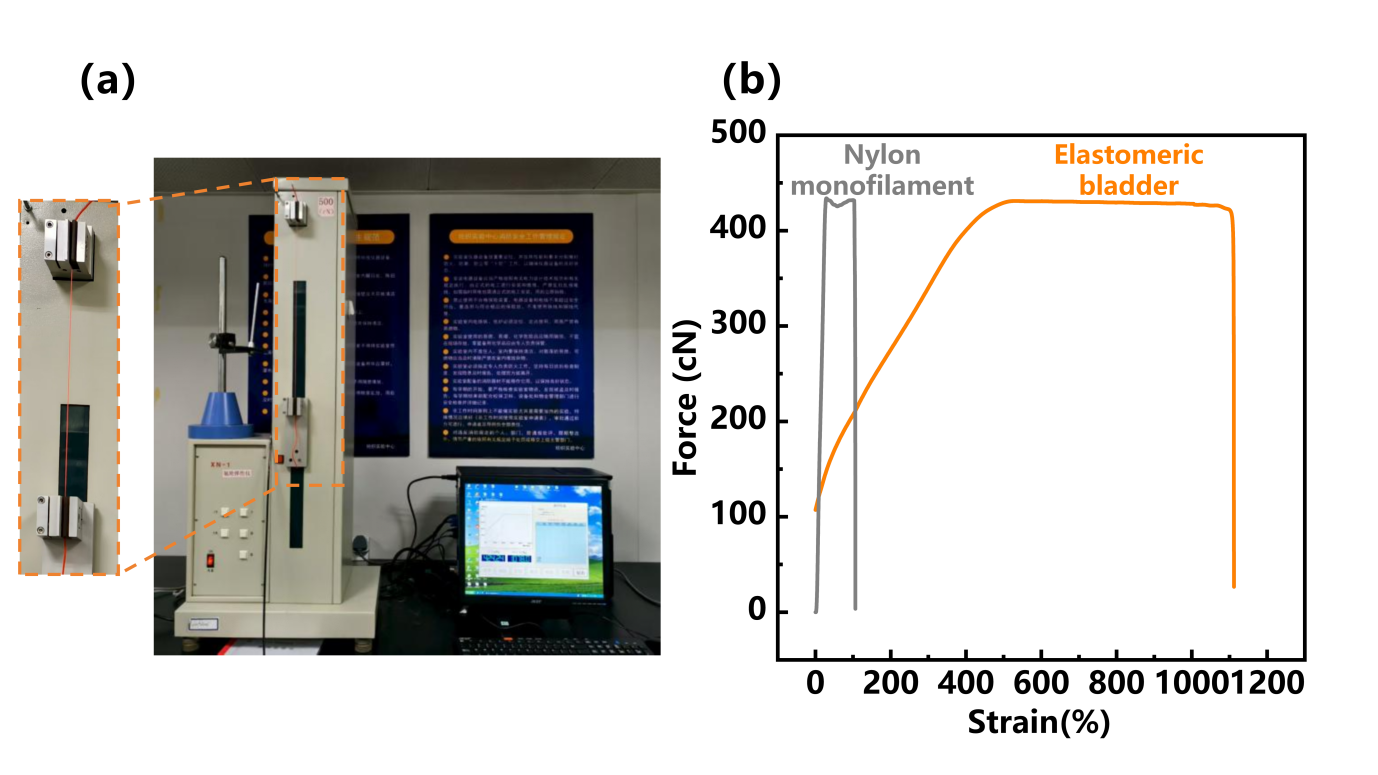


**Figure S3.** The mechanical properties of PET yarn and elastomeric bladder.

1. Yarn mechanical properties testing. (b) Stress-strain curves of nylon monofilament and elastomeric bladder. A 100 cN preload was applied to the hyperelastic bladder prior to measurement to accommodate instrument range limitations. Under a 400 cN load, the bladder exhibited over 1100% elongation, in contrast to only 100% for the nylon monofilament.


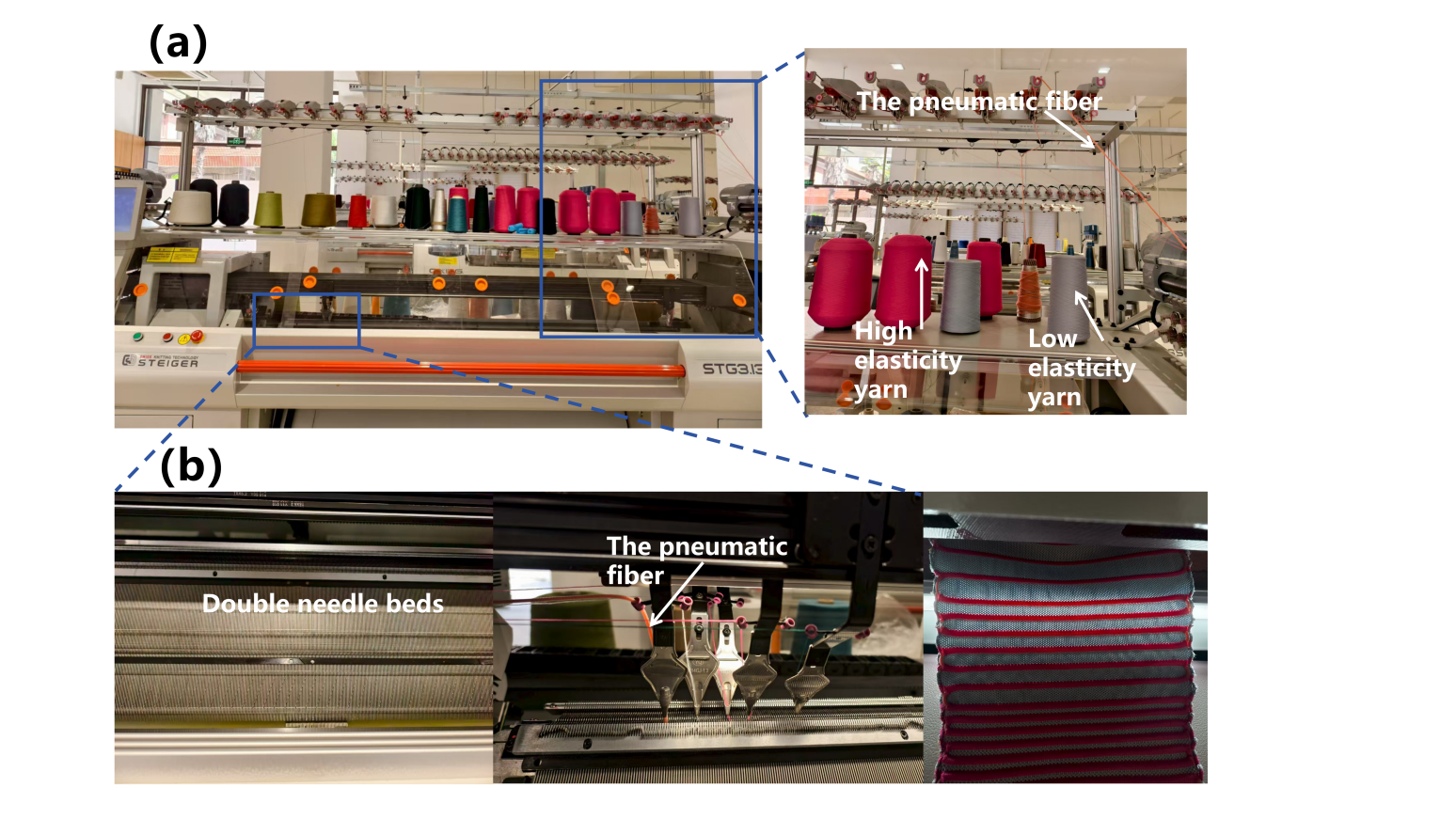


**Figure S4.** The textile production processes for the PPKF. (a) Double-needle-bed knitting machine. (b) Knitting details.


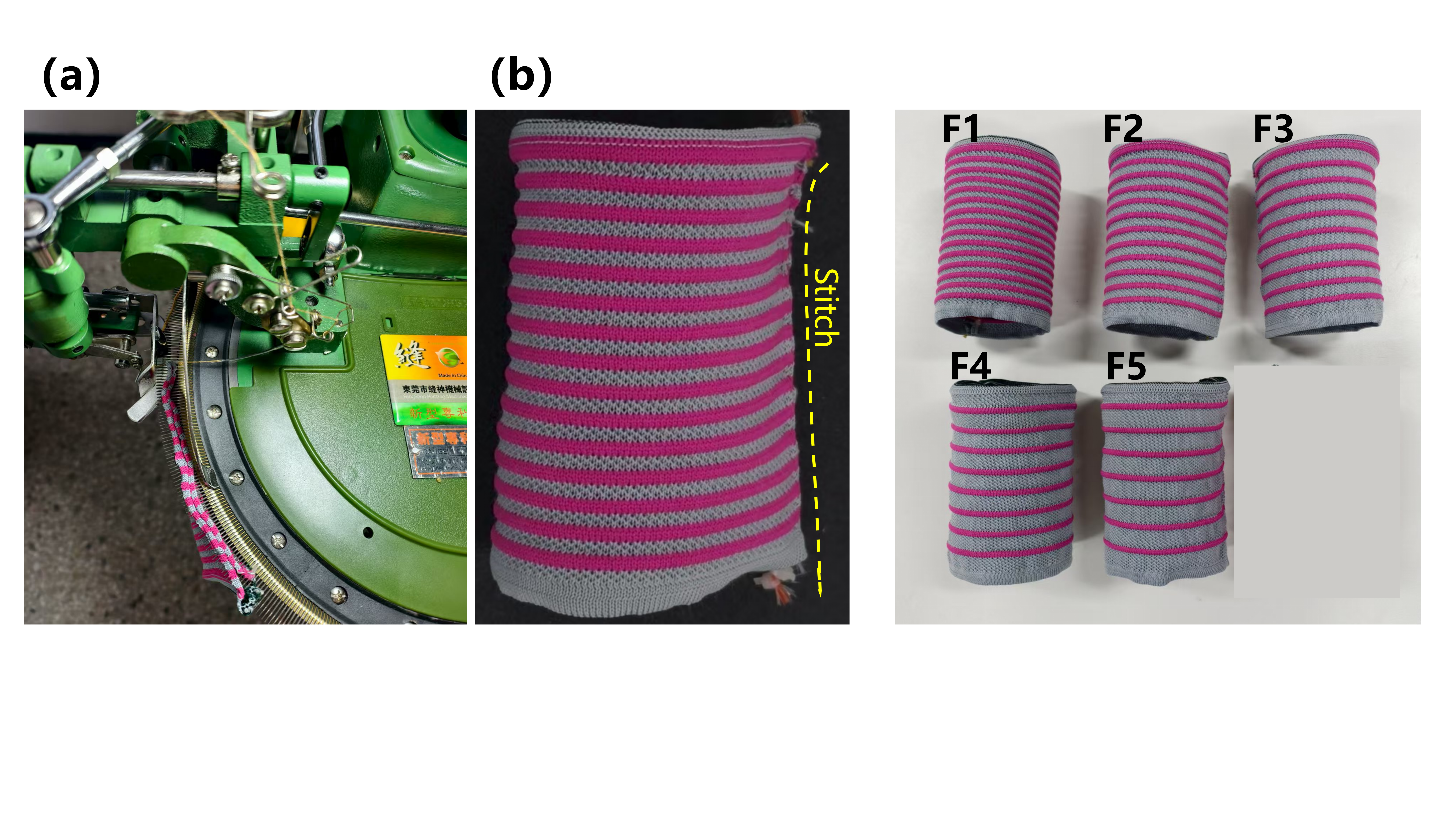


**Figure S5.** Fabrication of a 3D PPK. (a) DiallinKing machine. (b) The details.


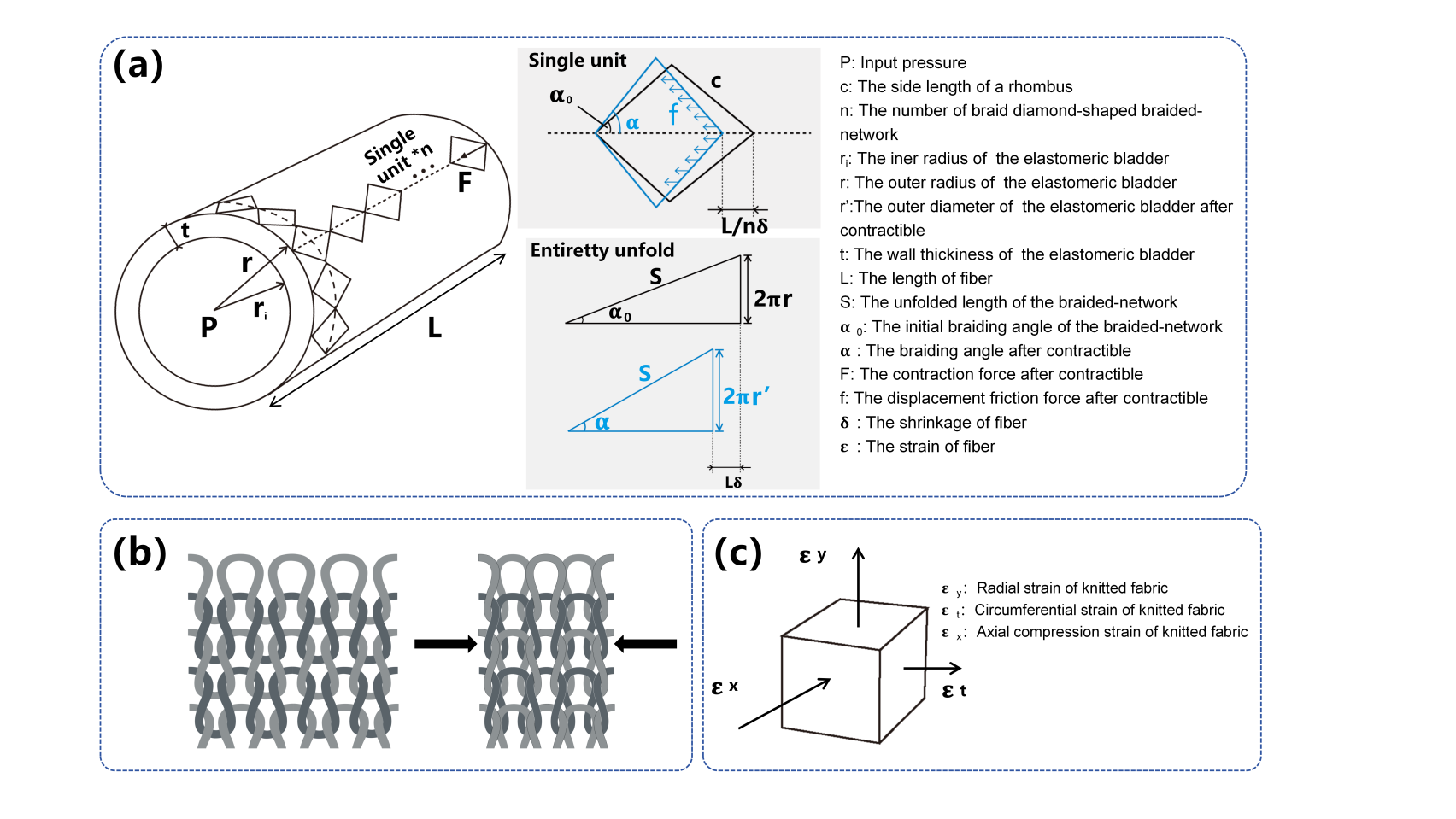


**Figure S6.** Mechanism of controllable compression in PPKF.

1. Pneumatic fiber contraction mechanism.

The fiber consists of *n* diamond-shaped braid meshes, upon internal pressurization *P*, the elastomeric bladder undergoes radial expansion, increasing its circumferential length while reducing the initial braiding angle from *α_0_* to *α.* Due to their high elastic modulus, the diamond-shaped reinforcing fibers maintain constant side length *c*. This geometric constraint generates axial contraction force *F* and displacement $\text{L/nδ}$, with frictional resistance *f* opposing deformation.

The outer helical reinforcement layer is modeled via right triangles:

Pre-inflation: Black triangle (baseline state)

Post-inflation: Blue triangle (deformed state)

While hypotenuse *S* remains constant, the vertical leg (representing fiber circumference *2πr*) increases with radial expansion. The combined action of braided-network contraction force *F* and deformation resistance *f* produces net axial contraction $L\delta$. Maximum contraction force occurs at critical braiding angle *αₘₐₓ*, enabling precise compression control.

1. Textile substrate coupling.

When pneumatically contracted fibers integrate with knitted substrates, axial deformation transfers directly to the textile layer. Assuming tight fiber-to-fabric coupling, substrate contraction equals fiber contraction, as the Equation S1:

$\text{∆}\text{L}_{\text{textile}}\text{=}{\text{∆}\text{L}}_{\text{fiber}}$ (S1)

1. Therapeutic pressure generation.

Modeling the textile as a constant-volume unit (neglecting circumferential strain $\text{ε}_{\text{t}}$), axial compression strain $\text{ε}_{\text{x}}$ equals radial strain $\text{ε}_{\text{y}}$ by volume conservation, as the Equation S2:

$\text{ε}_{\text{x}}\text{=}\text{ε}_{\text{y}}$ (S2)

This strain conversion generates surface pressure for therapeutic compression.


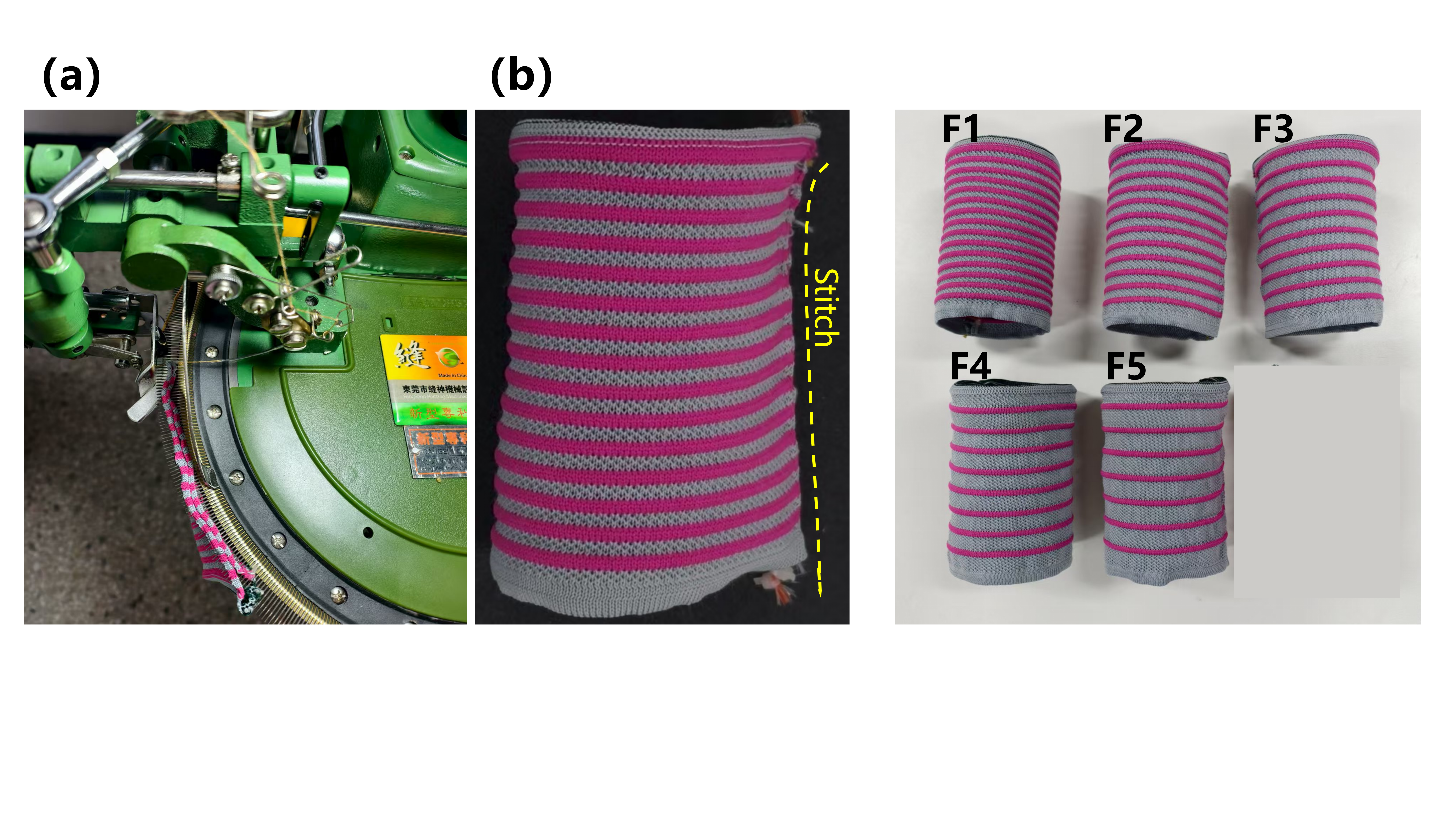


**Figure S7.** Five PPKF samples with different channel densities.


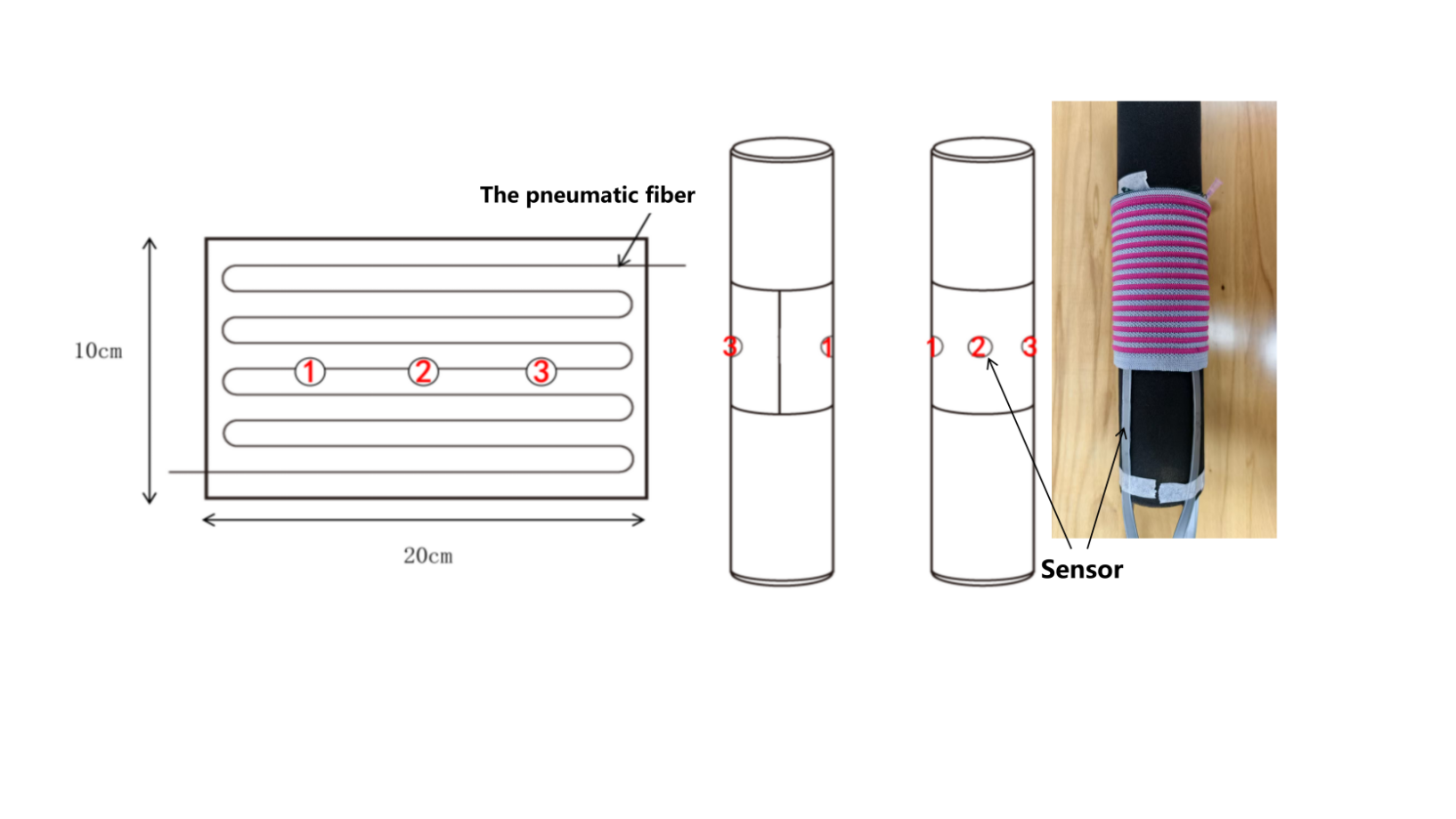


**Figure S8.** Deformation pressure test.

The PPKF was woven into a 200 mm × 100 mm rectangular shape. A 5.5 cm diameter acrylic cylinder was surfaced with a 0.3 cm-thick polyurethane foam to simulate human soft tissue. Pressure sensors were positioned at three non-seam locations, with recorded deformation pressures averaged across all sensors.


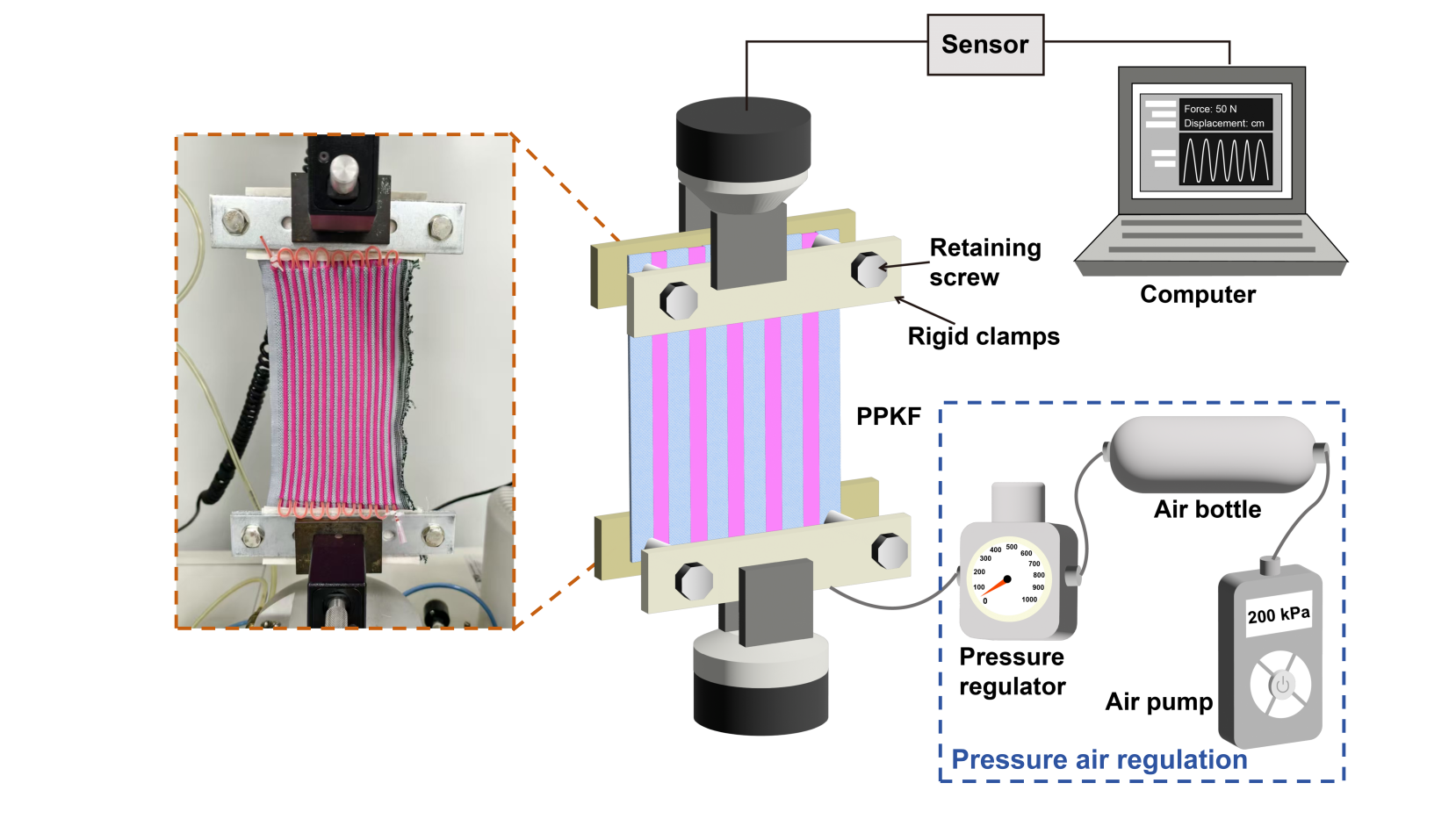


**Figure S9.** Contraction test.

Experimental Procedure:
Step 1: Apply 5 N pre-tension and zero the force sensor.
Step 2: Input target pressure (0-800 kPa), record sensor force *F*

(Accounting for clamp-induced forces).
Step 3: Displace clamps along contraction direction until sensor reads 0 N.
Step 4:Return clamps to initial position (zero displacement), record corrected force *F'*.
Step 5:Calculate final contraction force as mean *F'* value.


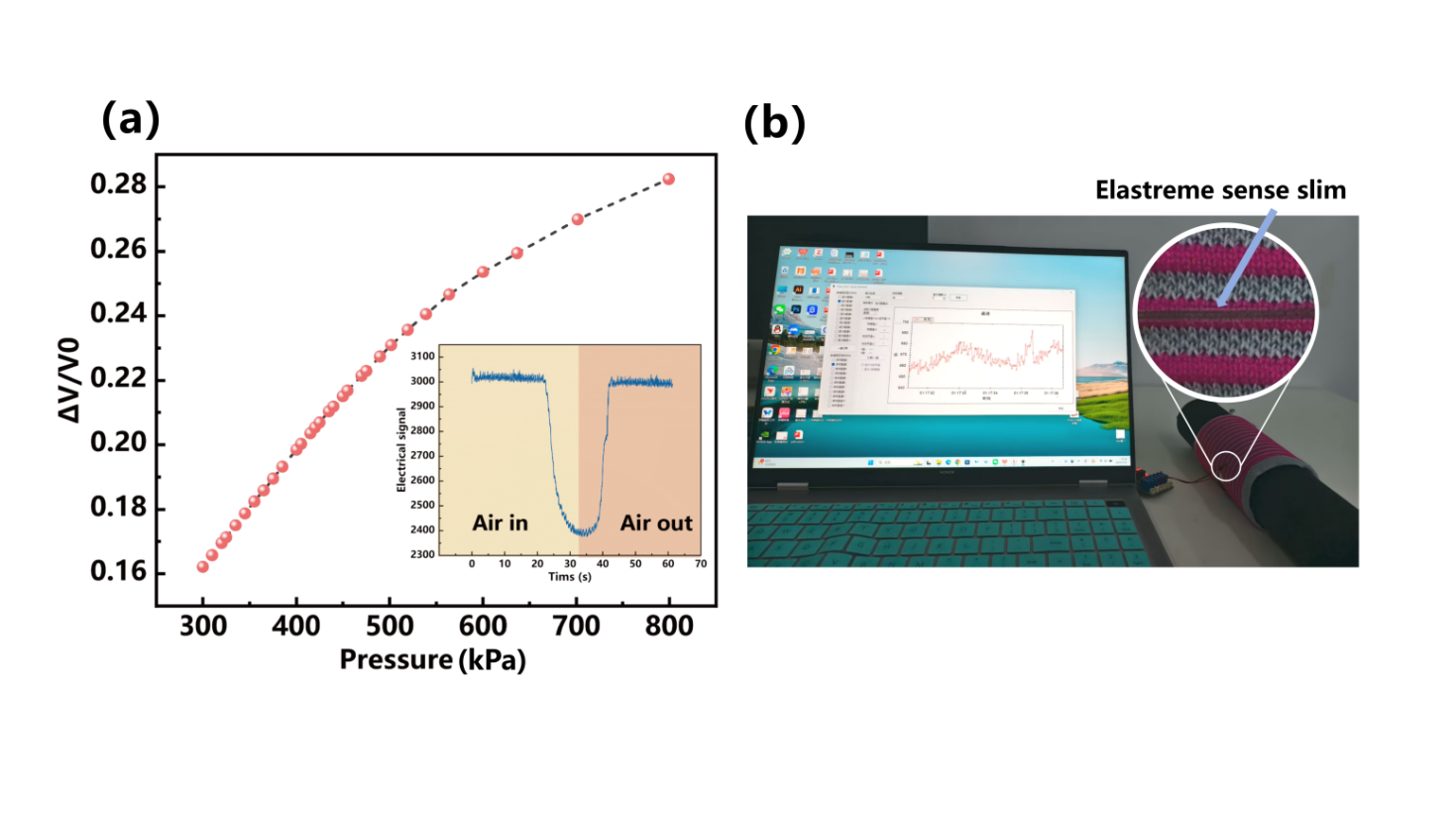


**Figure S10.** Real-time pressure feedback based on soft sensors (Elastreme sense slim).

1. The voltage rate of PPKF at 300-800 kPa. (b) Elastreme sense slim integrated into PPKF.


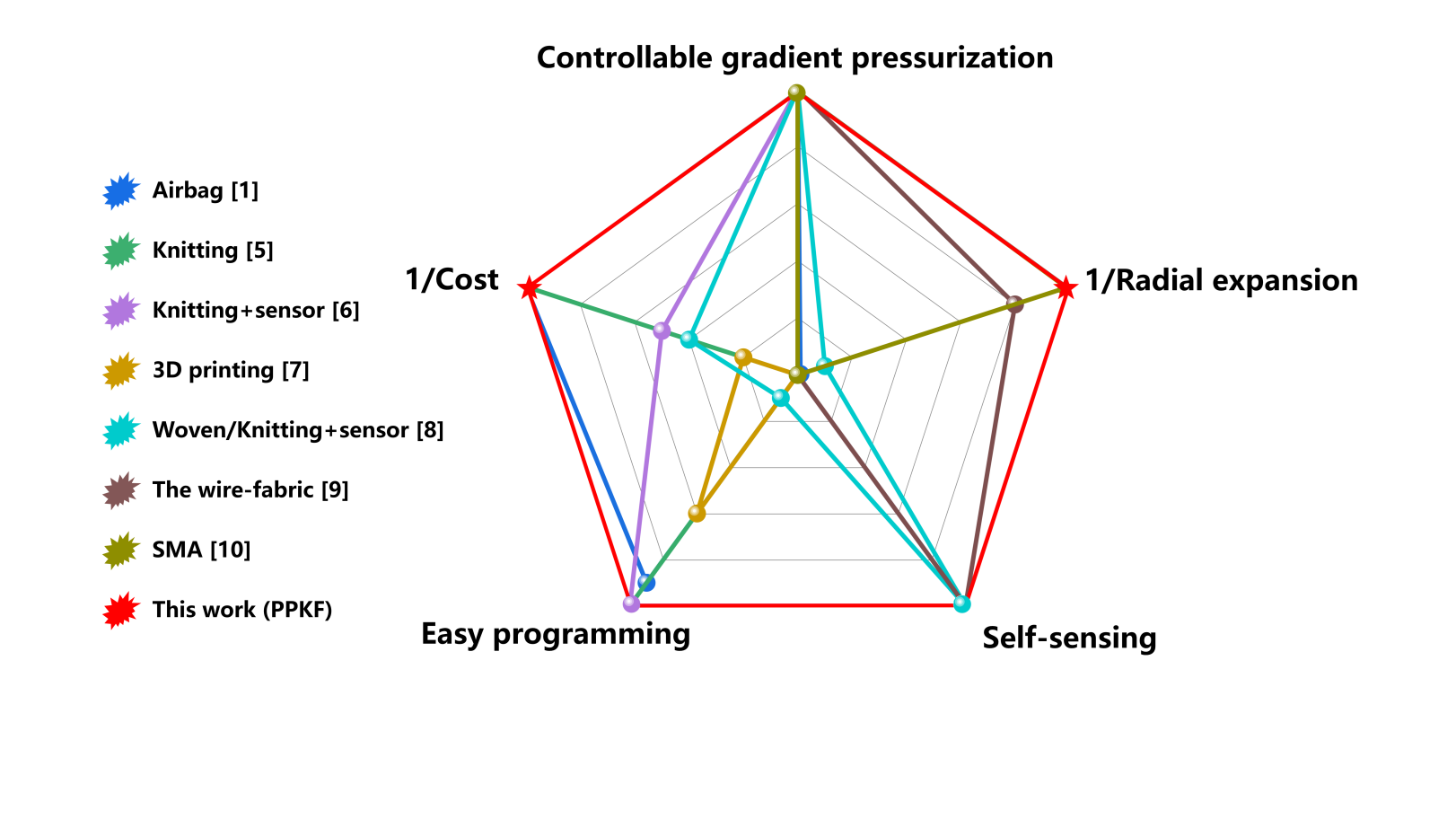


**Figure S11.** A comparative analysis of the PPKF with the main performance characteristics of current compression garments.


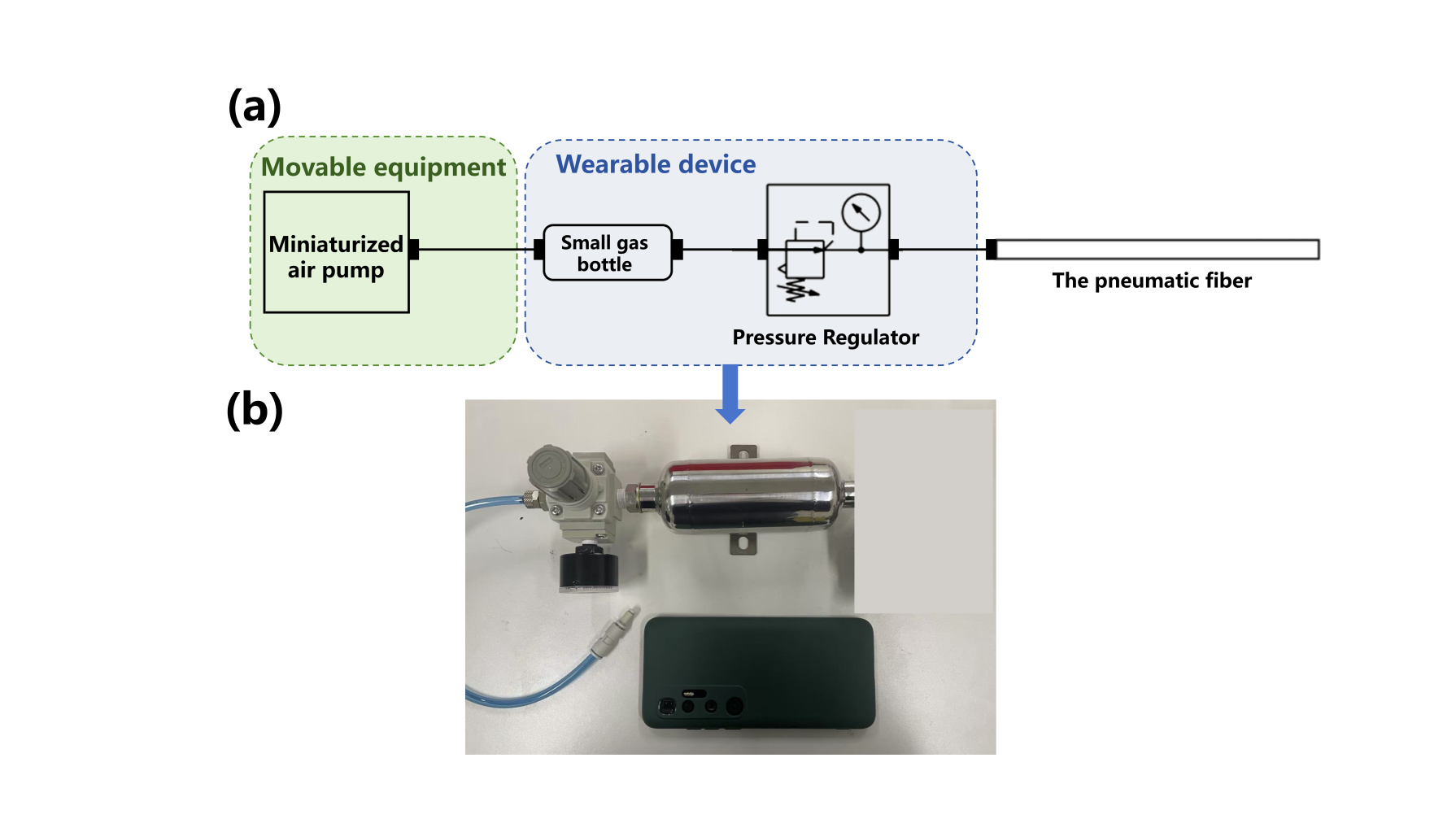


**Figure S12.** Wearable air supply equipment. (a) Design drawing. (b) The prototyping of equipment.


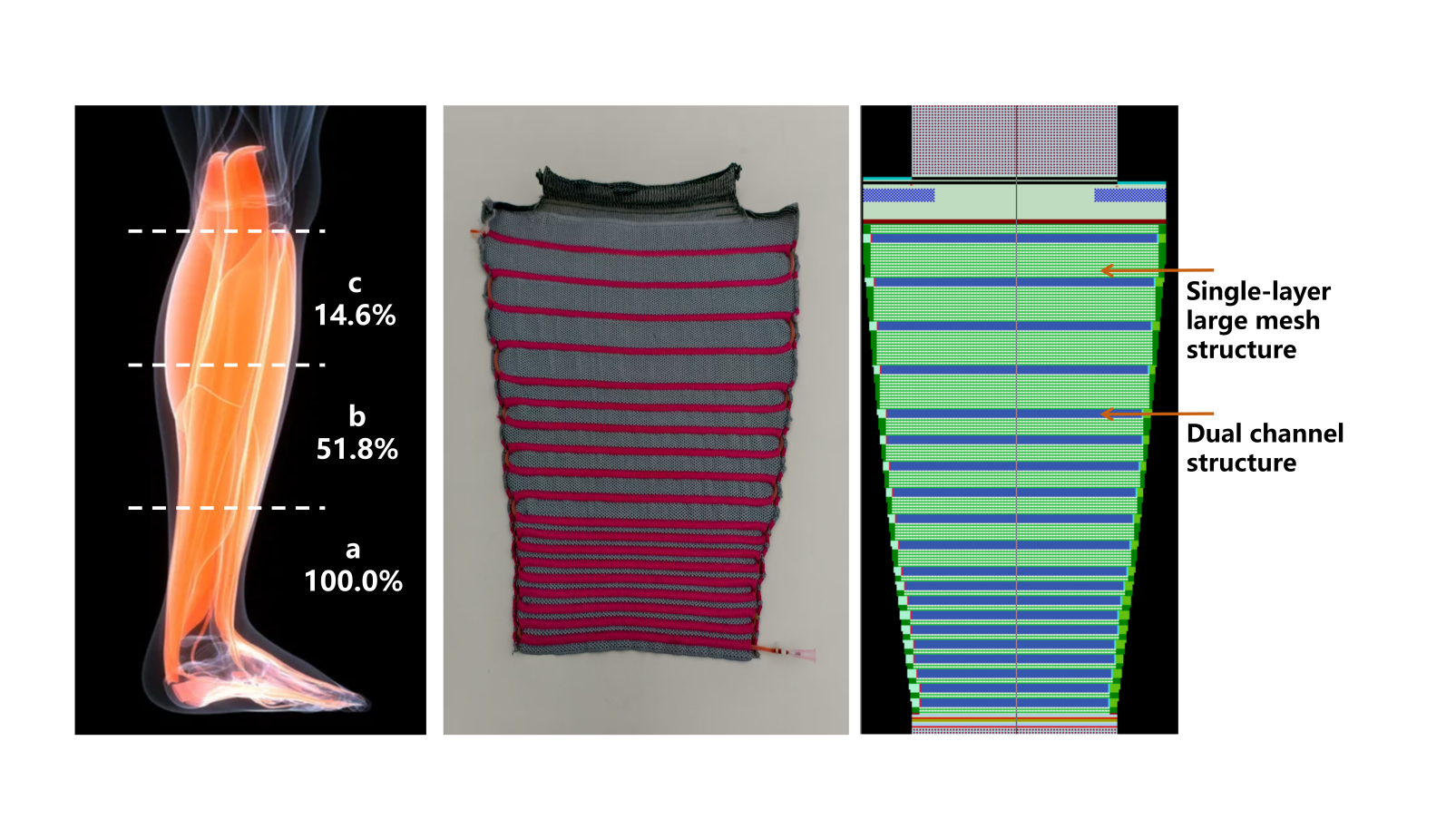


**Figure S13.** The gradient compression ratio and knitting pattern of the pneumatic gradient compression calf sleeve.

**
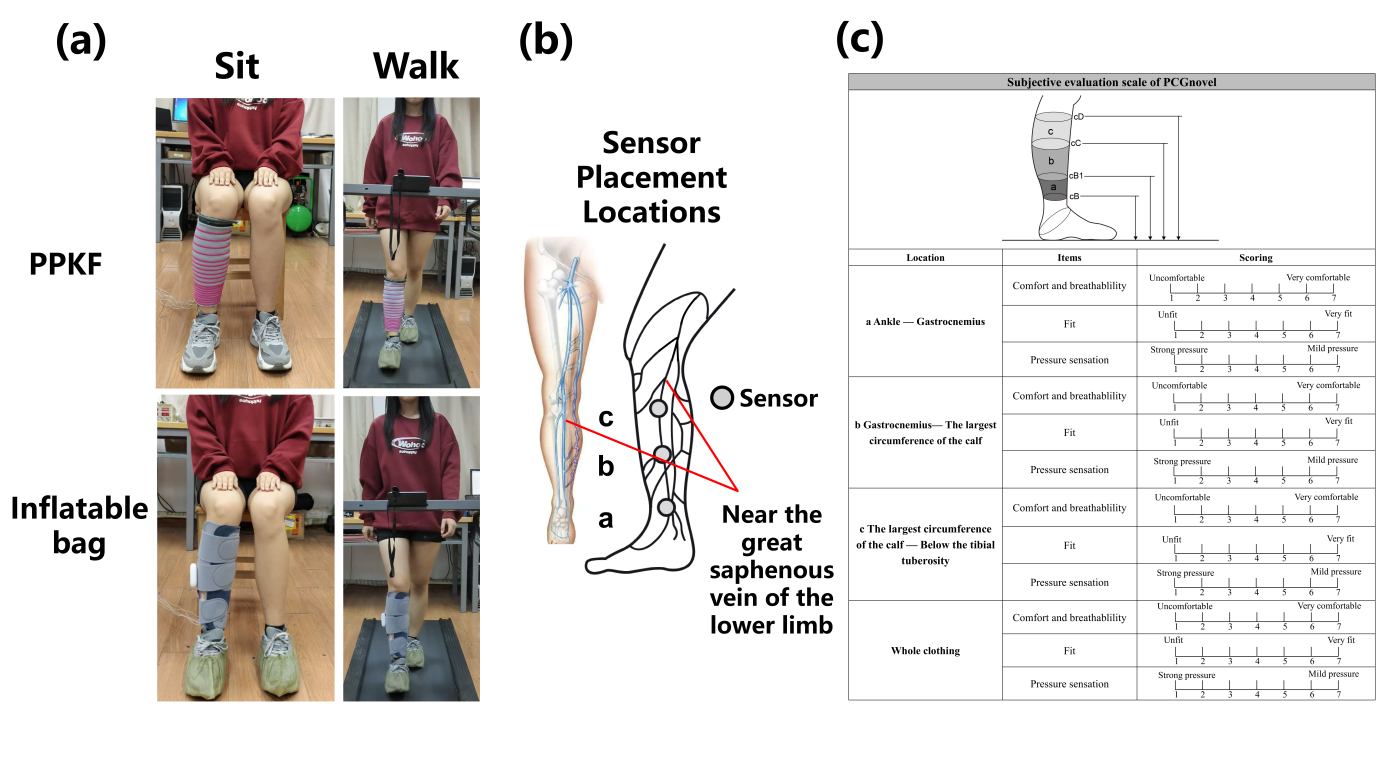
**

**Figure S14.** Objective pressure test and subjective rating evaluation. (a) The comparison of wearable garment: pneumatic gradient compression calf sleeve made of PPKF, commercial pneumatic compression garments made of inflatable bag (yht21A, Ningbo Haorou Medical Technology Co., Ltd., China). (b) Sensor placement locations.^[11]^ (c) Subjective rating scale.

**Supporting Table (Table S1-S2)**

**Table S1** Comparisons of PAM in current research

| **Reference** | **Diameter (mm)** | **Initial braiding angle** | **The quantity of yarn** | **Elastomeric bladder materials** |
| --- | --- | --- | --- | --- |
| Kurumaya et al [2] | 1.8 | 18$^{\circ}$ | 24 | Silicone |
| Hiramitstu et al [3] | 2.2 | 19$^{\circ}$ | 32 | Rubber |
| Marshall et al [4] | 3 | 19$^{\circ}$ | 32 | Rubber |
| **This work** | **2.5** | **20.83**$\boldsymbol{^{\circ}}$ | **24** | **Rubber** |

**Table S2** Comparisons of reported compression garments by ordinal numbering

| **Structure/Materials** | **Controllable gradient pressurization** | **Radial expansion** | **Self-**  **sensing** | **Easy programming** | **Cost** | **References** |
| --- | --- | --- | --- | --- | --- | --- |
| Airbag | 1 | 250.0% | 0 | L1 | C1 | [1] |
| Knitting | 0 | - | 0 | L1 | C1 | [5] |
| Knitting + Sensor | 1 | - | 1 | L1 | C3 | [6] |
| 3D printed | 0 | - | 0 | L2 | C2 | [7] |
| Woven/Knitting  + sensor | 1 | 162.0% | 1 | L3 | C3 | [8] |
| The wire - fabric | 1 | 8.5% | 1 | L3 | C3 | [9] |
| SMA | 1 | 4.3% | 0 | L3 | C3 | [10] |
| **PPKF** | **1** | **5.2%** | **1** | **L1** | **C1** | **This work** |

**Note:** In Table S2, “0” indicates that the actuator does not have the corresponding property, while “1” means that the actuator has the corresponding property. The data and assertions in

Table S2 are explained below:

**Radial expansion:**

The compressure garments radial expansion is defined by the diameter of the garments at its ratio of the final drive maximum diameter ($\text{D}_{\text{max}}$) minus the initial diameter (*D_inital_*) to the initial diameter, which is calculated by the Equation S3:

$\text{Radial expansion (\%)}\text{=}\frac{D_{max}-D_{inital}}{D_{inital}}$ (S3)

**Easy programming:**

The most common materials used for creating compression garments are knitted structure design based on human body zones, but controllable gradient pressure is usually not possible. Methods for weaving actively controllable compression garments include inflation and pressure, temperature-controlled fibers (SMA, metal wire, etc.), integrated sensors, etc., which are usually complex to manufacture and cannot be achieved through integrated weaving. By contrast, PAM-based pneumatic knitted compression garments can be easily adjusted by tuning structures and materials of the textile layer during manufacturing, making them easy to program. 3D printing is an emerging method for fabricating the actuators with different structures, but the narrow material adaptability of this method limits the versatile design for compression garments. Therefore, we can rank the programmability of the garments from Level 1 (L1, easy programming) to Level 3 (L3, difficult programming) based on the manufacturing methods: textile manufacturing (L1), 3D printing (L2), and assembling (L3).

**Cost:**

The cost involves two parts, namely, materials and manufacturing costs. The textile materials used for fabricating the fabric of the compression garments are primarily commercial synthetic materials including elastane yarns (ref. [5]), high-elastic polyester yarn (ref. [6] and our work), polyamide-elastane (ref. [8]). The cost of these yarns is reasonable and inexpensive, with prices ranging from $1.6/kg to $4.0/kg (according to the prices listed on www.alibaba.com, accessed in July 2025). The pneumatic fiber are generally fabricated by elastic rubber or silicone, with prices of ≈ $1.12/m^2^ - $2.09/m^2^ based on thickness (0.2 mm - 1.2 mm) and hardness (30 - 50 A). Moreover, conventional sensing sensors built-in the garments use expensive raw materials, such as ITO conductive membrane (≈ $1300/m^2^) and integrated flexible electronics. In addition, most of the shape memory alloys (SMA) that can be integrated in garments are NiTi, which is expensive with prices of ≈ $6/m^2^ - $15/m^2^; on the contrary, the low-cost conductive yarns, such as stainless-steel yarn (≈ $5/kg), graphene conductive yarn (≈ $40/kg) and silver-coated conductive yarn (≈ $40/kg), can be woven, knitted, braided or stitched into the textile layer of textile-based garments to form sensing region for sensing functions at a low-cost strategy.

Regarding manufacturing cost, the modern textile technologies enable easy fabrication of

the compression garments with programmed structures and mechanical properties. Moreover, the high scalability of the textile manufacturing process also contributes to the low cost. However, the manufacturing cost of the assembling processes is relatively high due to the complex molding and time-consuming for design garments with desired function and performance. The limited scalability of assembling and 3D printing methods also restrict the low-cost production of garments. Therefore, we can roughly rank the cost in three levels based on the materials and manufacturing costs: textile-based compression garments as C1 (lowest cost), printed compression garments as C2, and assembling compression garments as C3 (highest cost).

**Supporting Movies (Movie S1-S3)**

**Movie S1:** Contractible deformation of the pneumatic fiber.

**Movie S2:** The change in the braiding angle of the pneumatic fiber at 0 - 800 kPa.

**Movie S3:** The radial expansion of the inflatable bag and PPKF.

**Movie S4:** Contractible deformation of PPKF

**References for Supporting Information**

1. https://www.medicalexpo.com.cn/prod/fisiopress/product-4578784-1093684.html
2. S. Kurumaya, H. Nabae, G. Endo, et al., “Design of thin McKibben muscle and multifilament structure,” *Sensors and Actuators A: Physical* 261 (2017): 66-74. <https://doi.org/10.1016/j.sna.2017.04.047>
3. T. Hiramitsu, K. Suzumori, H. Nabae et al., “Experimental Evaluation of Textile Mechanisms Made of Artificial Muscles,” *in proceeding of 2nd IEEE International Conference on Soft Robotics* (2019).
4. R. Marshall, J. R. G. Souppez, M. Khan, et al., “Mechanical Characterisation of Woven Pneumatic Active Textile,” *IEEE Robotics and Automation Letters* 8, no. 5 (2023): 2804-2811. <https://doi.org/10.1109/LRA.2023.3262177>
5. I. Pita Miguélez, A. R. Labanieh, C. Mesmin, et al., “Development of a hybrid model of the tensile behaviour of weft-knitted structures for medical compression stockings,” *Journal of Engineered Fibers and Fabrics*, no. 20 (2025). <https://doi.org/10.1177/15589250251352034>
6. X. Zhang, Y. Tang, L. Shen, et al., “Optimal Design of Remote Monitoring Wearable Devices for Patients with Varicose Veins in Lower Extremities,” *ZHUANGSHI*, no . 11 (2020): 120-123. [https://doi.org/10.16272/j.cnki.cn11-1392/j.2020.11.035](https://doi.org/10.16272/j.cnki.cn11-1392/j.2020.11.035.)
7. J. Wang, J. G. Lu, and X. L. Cui, “Compression graments,” (2024). CN 110537746 A
8. C. J. Payne, E. G. Hevia, N. Phipps, et al., “Force control of textile-based soft wearable robots for mechanotherapy,” *in Proceedings of the 2018 IEEE International Conference on Robotics and Automation (ICRA)* (2018): 5459-5465. <https://doi.org/10.1109/ICRA.2018.8461059>
9. S. T. Yang, J. W. Ryu, S. H. Park, et al., “An active compression sleeve with variable pressure levels using a wire-fabric mechanism and a soft sensor,” *Smart Materials and Structures*, 28, no. 11 (2019): 114002. <https://doi.org/10.1088/1361-665X/ab3f56>
10. R. Pettys-Baker, R. Granberry, N. Subash, et al., “Wearability and Comfort Improvements to Active Compression Stockings for Lower Leg Compressive Therapy,” *in Proceedings of the 2022 ACM International Symposium on Wearable Computers (ISWC '22).* (2022): 127-130. <https://doi.org/10.1145/3544794.3560812>
11. N. Labropoulos, A. D. Giannoukas, K. Delis, et al., “The impact of isolated lesser saphenous vein system incompetence on clinical signs and symptoms of chronic venous disease,” Journal of vascular surgery, 32 no. 5 (2000): 954-960.
